# DAF-16/FOXO promotes taste avoidance learning independently of axonal insulin-like signaling

**DOI:** 10.1371/journal.pgen.1008297

**Published:** 2019-07-19

**Authors:** Takashi Nagashima, Yuichi Iino, Masahiro Tomioka

**Affiliations:** Department of Biological Sciences, Graduate School of Science, The University of Tokyo, Tokyo, Japan; Princeton, UNITED STATES

## Abstract

The avoidance of starvation is critical for the survival of most organisms, thus animals change behavior based on past nutritional conditions. Insulin signaling is important for nutritional state-dependent behavioral plasticity, yet the underlying regulatory mechanism at the cellular level remains unclear. Previous studies showed that insulin-like signaling is required for taste avoidance learning, in which the nematode *Caenorhabditis elegans* avoids salt concentrations encountered under starvation conditions. DAF-2c, a splice isoform of the DAF-2 insulin receptor, functions in the axon of the ASER sensory neuron, which senses changes in salt concentrations. In addition, mutants of a major downstream factor of DAF-2, the forkhead transcription factor O (FOXO) homolog DAF-16, show defects in taste avoidance learning. Interestingly, the defect of the *daf-2* mutant is not suppressed by *daf-16* mutations in the learning, unlike those in other phenomena, such as longevity and development. Here we show that multiple DAF-16 isoforms function in ASER. By epistasis analysis using a DAF-2c isoform-specific mutant and an activated form of DAF-16, we found that DAF-16 acts in the nucleus in parallel with the DAF-2c-dependent pathway in the axon, indicating that insulin-like signaling acts both in the cell body and axon of a single neuron, ASER. Starvation conditioning induces nuclear translocation of DAF-16 in ASER and degradation of DAF-16 before starvation conditioning causes defects in taste avoidance learning. Forced nuclear localization of DAF-16 in ASER biased chemotaxis towards lower salt concentrtions and this effect required the Gq/PKC pathway and neuropeptide processing enzymes. These data imply that DAF-16/FOXO transmits starvation signals and modulates neuropeptide transmission in the learning.

## Author summary

Animals change behavior based on remembered experiences of hunger and appetite. Signaling by insulin and insulin-like peptides in the nervous system plays key roles in behavioral responses to hunger and satiety. In *C*. *elegans*, insulin-like signaling in the gustatory sensory neuron ASER regulates learned avoidance of salt concentrations experienced during fasting, which we call taste avoidance learning. DAF-2c, an isoform of the insulin receptor homolog, is localized to the axon of ASER and regulates taste avoidance learning. Here, we show that DAF-16, the forkhead transcription factor O (FOXO) homolog, translocates into the nucleus of ASER during fasting and promotes taste avoidance learning. DAF-16 is negatively regulated by insulin-like signaling independently of axonal DAF-2c signaling. This dual function of insulin-like signaling in the cell body and the axon ensures dynamic changes in behavioral responses after experience of hunger. By genetic analyses using constitutively nuclear-translocated DAF-16, we show that DAF-16 in ASER regulates taste avoidance learning via modulating neuropeptide signaling in the nervous system, which is reminiscent of the function of FOXO in the hypothalamus in the regulation of food-seeking behavior in mammals.

## Introduction

A common strategy for animals to respond to starvation conditions is to change behaviors based on the nutritional state. Insulin/IGF signaling plays a key role in physiological and behavioral responses to nutritional stimuli. For instance, insulin signaling is involved in nutritional condition-dependent regulations of metabolism, development and longevity [1,2]. Insulin signaling acting in the nervous system controls feeding behavior and its dysfunction leads to neurological disorders [3,4]. Neural insulin signaling also affects valuation of nutritional stimuli in the human brain [5]. However, the functions of insulin signaling in the central nervous system, especially at the single-neuron level, have not been well clarified.

*Caenorhabditis elegans* is a commonly used model to investigate behavioral plasticity because of its ease of genetic manipulation and analyses at the single cell level *in vivo*. Most components of insulin-like signaling in *C*. *elegans* are conserved across species. *C*. *elegans* has only one insulin receptor homolog, DAF-2, despite having 40 insulin-like peptides [6,7]. Downstream of the DAF-2 receptor, a signaling pathway composed of the phosphatidylinositol 3-kinase homolog AGE-1 [8], the 3-phosphoinositide-dependent kinase-1 homolog PDK1 and the Akt/PKB homologs AKT-1/2 [9,10] negatively regulates the forkhead transcription factor O (FOXO) homolog DAF-16 [11–14]. DAF-16 is expressed in various tissues and controls several phenomena, such as stress response, metabolism, dauer formation, and lifespan [15–17]. Recently, tissue-specific transcriptome analyses showed that the regulation of gene expression by DAF-16 differs between the nervous system and other tissues [18,19]. DAF-16 function in AWB chemosensory neurons contributes to pheromone-dependent behavior via transcriptional control of the *glna-3* gene, which encodes a glutaminase homolog [20]. Moreover, DAF-2/DAF-16 signaling is required for behavioral plasticity. DAF-16 regulates starvation-dependent increase in pheromone repulsion in the ADL chemosensory neurons, as well as starvation-dependent thermotaxis plasticity in the thermosensory circuit [21–23]. DAF-16 enters the nucleus dependent on nutritional conditions and DAF-2 signaling [11]. However, it is unclear whether DAF-2/DAF-16 signaling mediates transmission of nutritional states required for the formation of memory in the regulation of the feeding status-dependent behavioral plasticity.

*C*. *elegans* worms migrate toward sodium chloride concentrations experienced during feeding, but avoid such concentrations experienced during fasting, in a phenomenon called taste avoidance learning [24,25]. We reported that insulin-like signaling is required for taste avoidance learning and acts in the salt-sensing neuron, ASER [25,26]. Isoform-specific axonal transport of the DAF-2 isoform DAF-2c is required for taste avoidance learning. Starvation increases translocation of DAF-2c from the cell body to the axon, and this translocation is carried by kinesin-1-dependent axonal transport via the cargo adapter CASY-1, a homolog of mammalian Calsyntenin. DAF-2c/PI3K signaling decreases diacylglycerol (DAG) dynamics in the axon [27]. As high or low DAG levels promote animals’ migration toward high or low-salt concentration, respectively, axonal insulin-like signaling has been proposed to control the direction of salt chemotaxis from attraction to avoidance, at least partly, through the regulation of DAG dynamics. We demonstrated that the loss of DAF-16 causes impaired taste avoidance learning, similar to that of insulin-signaling mutants [26,28]. Although phenotypes of insulin-signaling mutants were suppressed by loss-of-function (lf) mutation of *daf-16* in behavioral plasticity in odor chemotaxis and thermotaxis [22,23], defects of insulin-signaling mutants in taste avoidance learning were not suppressed by *daf-16*(lf) mutations, implying that the role of DAF-16 may be different among different paradigms of behavioral plasticity.

Here, we further investigated the function of DAF-16 and found that multiple DAF-16 isoforms function in the ASER sensory neuron independently of axonal insulin-like signaling in taste avoidance learning. Using the auxin-inducible degradation system, we showed that DAF-16 is required around the time of taste avoidance learning, rather than during development. DAF-16 localized to the nucleus of ASER under starvation conditions. A mutant form of DAF-16, in which the putative Akt phosphorylation sites were mutated, strongly localized to the nucleus of ASER even in the presence of food. The forced nuclear localization of DAF-16 biased chemotaxis toward lower salt concentrations even after high-salt conditioning with food, and this effect was also observed in the mutant background of axonal insulin-like signaling. Thus, these findings suggest that different insulin-like signaling pathways work in the nucleus and the axon of ASER to control salt chemotaxis. Furthermore, we showed that the DAF-16-dependent salt avoidance requires the Gq/PKC signaling and neuropeptide-processing pathways in the nervous system.

## Results

### DAF-16/FOXO is required for taste avoidance learning

**Fig 1:**
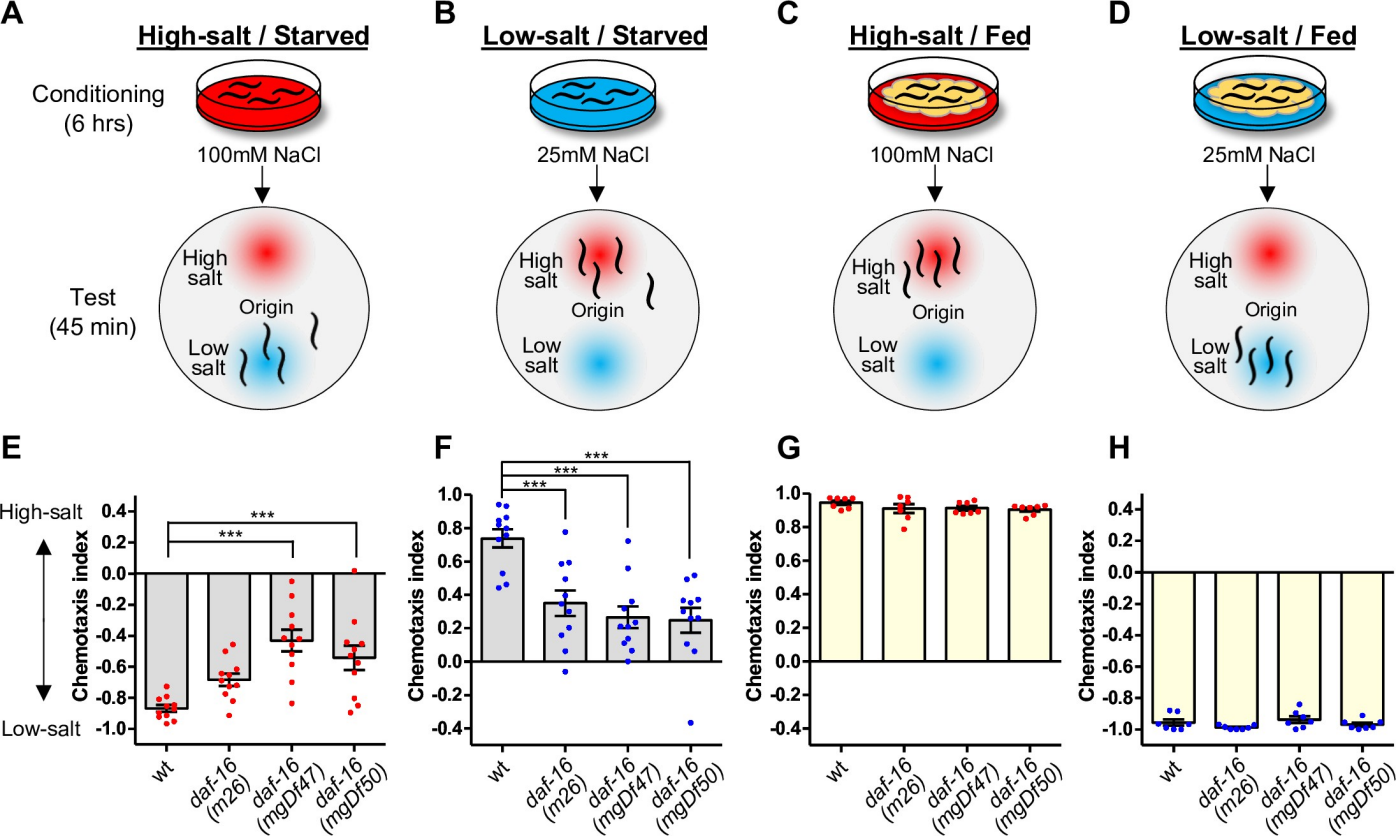
DAF-16/FOXO is required for taste avoidance learning. (A-D) Schematic of salt chemotaxis plasticity in *C*. *elegans*. After animals were conditioned on agar plates at high or low salt concentrations in the absence of food for six hours, they avoid the salt concentrations experienced under starvation (A, B). After animals were conditioned on agar plates at high or low salt concentrations in the presence of bacterial food, they migrate toward the salt concentrations encountered during feeding (C, D). (E-H) Salt chemotaxis of wild-type and *daf-16* mutant animals after conditioning with high- (E, G) or low-salt (F, H) in the absence (E, F) or presence (G, H) of food. Each dot in red or blue represents a chemotaxis index obtained from each trial when animals were conditioned at a high or low concentration of salt, respectively. Error bars indicate SEM. ANOVA with Dunnett’s test compared to wild type, ***p < 0.001. (E, F) N = 11. (G, H) N = 7.

Wild-type N2 animals avoid concentrations of sodium chloride (hereafter referred to as salt) encountered under starvation conditions, which is known as taste avoidance leaning [25]. After conditioning on agar plates that contain high or low concentrations of salt in the absence of food, adult animals migrate to areas of low or high salt concentrations, respectively, on a test plate with a salt gradient (Fig 1A, 1B, 1E and 1F). We first examined the requirement of DAF-16/FOXO in taste avoidance learning using three different *daf-16*(lf) mutants. *daf-16(mgDf47)* and *daf-16(mgDf50)* mutants showed weaker migration to low salt than wild type after starvation conditioning with high salt. The *daf-16(m26)* mutant exhibited a tendency of decreased low-salt migration after starvation conditioning, though the effect was not statistically significant (Fig 1E). Both *daf-16(mgDf47)* and *daf-16(mgDf50)* mutants harbor large deletions in the forkhead domains of all DAF-16 isoforms (WormBase), whereas the *b* isoform is spared in the *daf-16(m26)* mutant, suggesting that the weaker defect in *daf-16(m26)* mutant was likely attributed to the intact *b* isoform. After starvation conditioning with low salt, each of the *daf-16* mutants showed significant defects in high-salt migration, as compared to wild-type animals. These results indicate that DAF-16 is required for avoidance of high or low salt concentrations after starvation conditioning.

**Fig 2:**
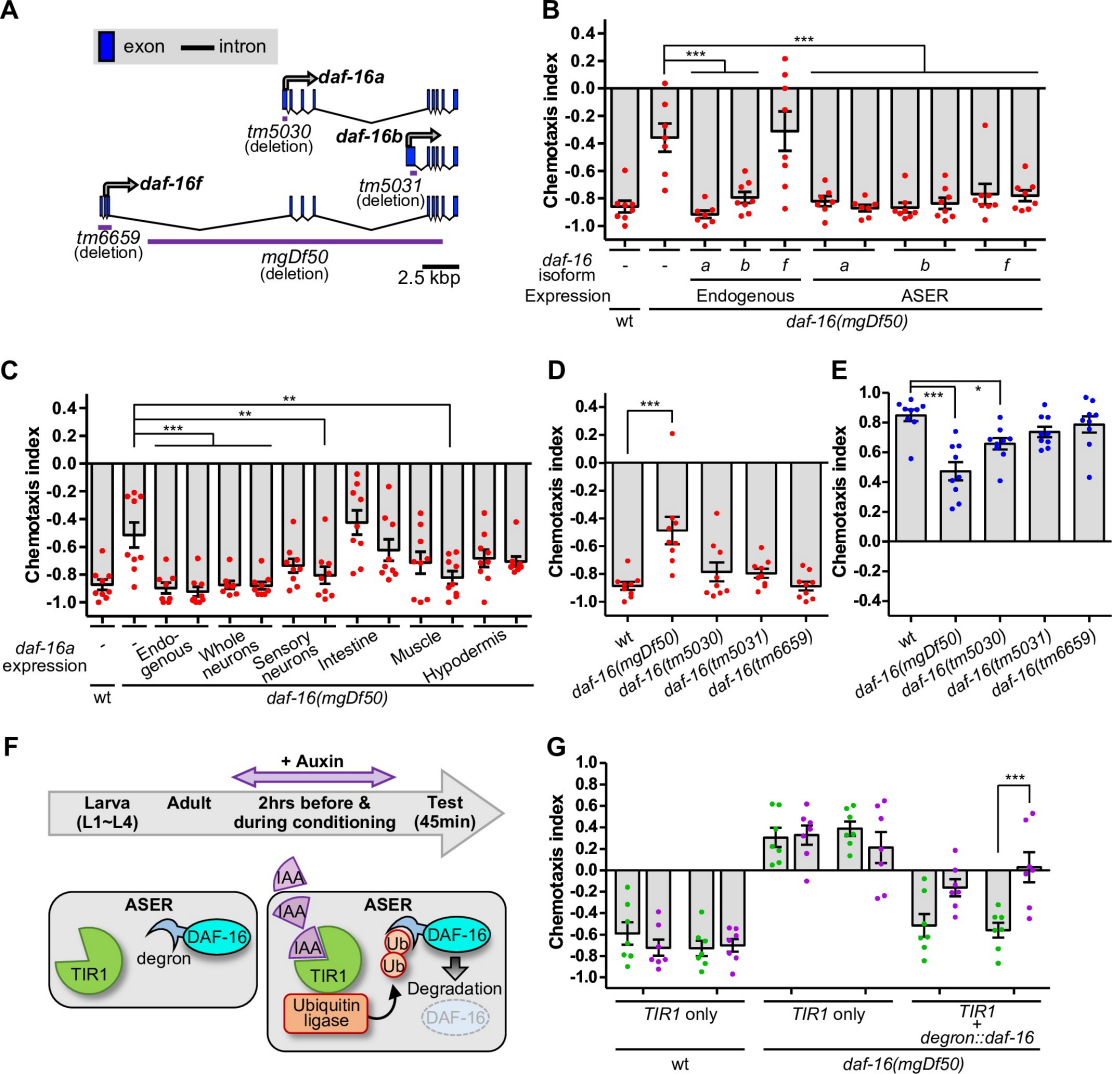
DAF-16 isoforms act in ASER during taste avoidance learning. (A) The genomic structures of DAF-16 isoforms and mutation sites of deletion mutants are shown. (B, C) Salt chemotaxis after high-salt conditioning in the absence of food. Each dot in red represents a chemotaxis index obtained from each trial. Each *daf-16* isoform cDNA was expressed in *daf-16(mgDf50*) mutant animals by its own promoter or the ASER-specific *gcy-5* promoter (B; N = 7 or 8). DAF-16a isoform was expressed by the *H20*, *odr-4*, *ges-1*, *myo-3*, and *dpy-7* promoter, which drove expression in most or all neurons, amphid and phasmid sensory neurons except ASE and AFD, intestine, muscle, and hypodermis, respectively (C; N = 8 or 9). (D, E) Salt chemotaxis of *daf-16* mutants carrying deletions in isoform-specific exon after high- (D) or low-salt (E) conditioning without food (N = 9). (A, B, D, E) Error bars indicate SEM. Dunnett’s test following one-way ANOVA, *p < 0.05, **p < 0.01, and ***p < 0.001. (F) Schematic of auxin-dependent expression of DAF-16 in ASER using degron-tagged DAF-16 and auxin interactive protein (TIR1). Auxin (IAA) induces ubiquitin (Ub)-dependent degradation of degron-tagged DAF-16. Animals are grown to the adult without auxin and then auxin was added for two hours before conditioning and during conditioning. (G) Salt chemotaxis of animals carrying transgenes after high-salt conditioning in the absence of food (N = 7). A green or purple dot represents a chemotaxis index obtained from each trial when treated with either the solvent, 0.25% ethanol or 1 mM auxin, respectively. Two lines of transgenic animals were tested. Error bars indicate SEM. Bonferroni multiple test following two-way ANOVA, ***p < 0.001.

On the other hand, the *daf-16* mutants showed no strong defect in associative learning between food and salt concentration: as the attraction to salt concentrations encountered during feeding was similar to that of the wild type (Fig 1C, 1D, 1G and 1H), although the *daf-16* mutation occasionally caused weak but significant reductions in high salt migration (S3A Fig) [24]. These results suggest that DAF-16 may also play a minor role in attractive behavior to high salt concentrations after feeding conditioning, but is particularly important for taste avoidance learning. They also suggest that a defect in taste avoidance learning does not appear to be due to a general defect in salt sensation or locomotion during chemotaxis. The *mgDf50* allele was mainly used for the following experiments because it had the largest deletion among the *daf-16* alleles (Fig 2A).

### Multiple DAF-16 isoforms function in the ASER neuron in taste avoidance learning

Multiple isoforms are generated from the *daf-16*-genomic region by alternative promoters and contain different N-terminal regions (Fig 2A). The *daf-16a* isoform, which has a sequence most similar to that of the mammalian FOXO3 among other isoforms, is involved in development, longevity, and stress responses [29]. The *daf-16b* isoform, which lacks a part of the forkhead domain, has not been reported to have a strong effect on these biological phenomena. The function of the *daf-16f* isoform is reported to affect longevity. We generated *daf-16-*rescue strains, each of which expressed a single *daf-16* isoform, as well as a fluorescent protein (Venus), under the endogenous promoter. As reported previously [12,29], the promoters of the *a*, *b*, and *f* isoforms drove expression in the whole body except for the pharynx, a few tissues, and almost all tissues except the gonad, respectively (S1 Fig). We also confirmed Venus expression in head neurons including ASER, by these promoters (S1H–S1J Fig). Next, we examined the learning ability of the *daf-16*-rescue strains. The defect of low-salt migration after starvation conditioning was canceled by expression of DAF-16a or DAF-16b, which was driven by each endogenous promoter in the *daf-16* mutant (Fig 2B), while no substantial effect was observed for DAF-16f (Fig 2B, S2A Fig). Among the DAF-16 isoforms, only DAF-16a expression significantly rescued high-salt migration after starvation conditioning in the *daf-16* mutant (S2B Fig). Because the rescue effect of DAF-16a was strongest, we performed tissue-specific rescue experiments of the *daf-16* mutant using *daf-16a* cDNA. Neuronal expression of DAF-16a fully rescued the learning defect of *daf-16* mutants (Fig 2C, S2C Fig). DAF-16a expression in ASER, but not in ASEL, was sufficient for full rescue of the low-salt migration defect (Fig 2B, S2D Fig) and partial rescue of the high-salt migration defect (S2B Fig), suggesting that DAF-16 acts in ASER for low-salt migration and in multiple neurons including ASER for high-salt migration after starvation conditioning. ASER expression of the *b* and *f* isoforms also rescued the learning defect of the *daf-16* mutant similar to that of the *a* isoform (Fig 2B, S2B Fig). These results suggest that all *daf-16* isoforms can function in ASER in taste avoidance learning. DAF-16a expression in multiple chemosensory neurons other than ASER or muscle cells was also sufficient for weak rescue of the learning defect (Fig 2C, S2C Fig), suggesting that DAF-16a can regulate taste avoidance learning also in multiple cell types other than ASER. We next examined the isoform-specific *daf-16* mutants, which were previously generated and were used for the study of longevity [30]. The *daf-16(tm5030)*, *daf-16(tm5031)*, and *daf-16(tm6659)* mutants, which harbor a deletion in the *a*, *b*, and *h/f/d* isoform-specific exon, respectively (Fig 2A), had no strong defects in low-salt migration, while the *daf-16(tm5030)* mutant had a mild defect in high-salt migration after starvation conditioning (Fig 2D and 2E). These results are consistent with the conclusion that taste avoidance learning is regulated by multiple DAF-16 isoforms with a strong contribution of DAF-16a.

### DAF-16 functions around the time of taste avoidance learning

DAF-16 is reportedly required for the development of AIY interneurons [31], whereas it functions in associative learning during the adult stage [18]. We investigated when DAF-16 functions in the regulation of taste avoidance learning, namely either during development or during the learning paradigm. We used the auxin-inducible degradation system, which is suitable for spatiotemporal analyses in several organisms including *C*. *elegans* [32,33]. In this system, two proteins are expressed in the same cells: one is a target protein tagged with the degron sequence that induces the ubiquitin-proteasomal degradation of the target protein and another is TIR1, a plant-specific F-box protein required for the activation of an E3 ubiquitin ligase. To control DAF-16 expression in a spatiotemporal manner, both degron-tagged DAF-16a and TIR1 were expressed in the ASER neuron of the *daf-16* mutant (Fig 2F). The transgenic animals were treated with 1 mM auxin (or 0.25% ethanol as a control) for two hours before conditioning and during conditioning with high salt. The low-salt migration after starvation conditioning was recovered by expression of degron-tagged DAF-16 in the *daf-16* mutant, and this recovery of low-salt migration was inhibited by auxin administration (Fig 2G). On the other hand, only TIR1 expression and/or auxin administration had no significant effect on chemotaxis of the wild-type and *daf-16* mutant animals. These results suggest that DAF-16 contributes to taste avoidance learning around the time of taste avoidance learning.

### DAF-16 functions in parallel with axonal insulin-like signaling in the ASER neuron

**Fig 3:**
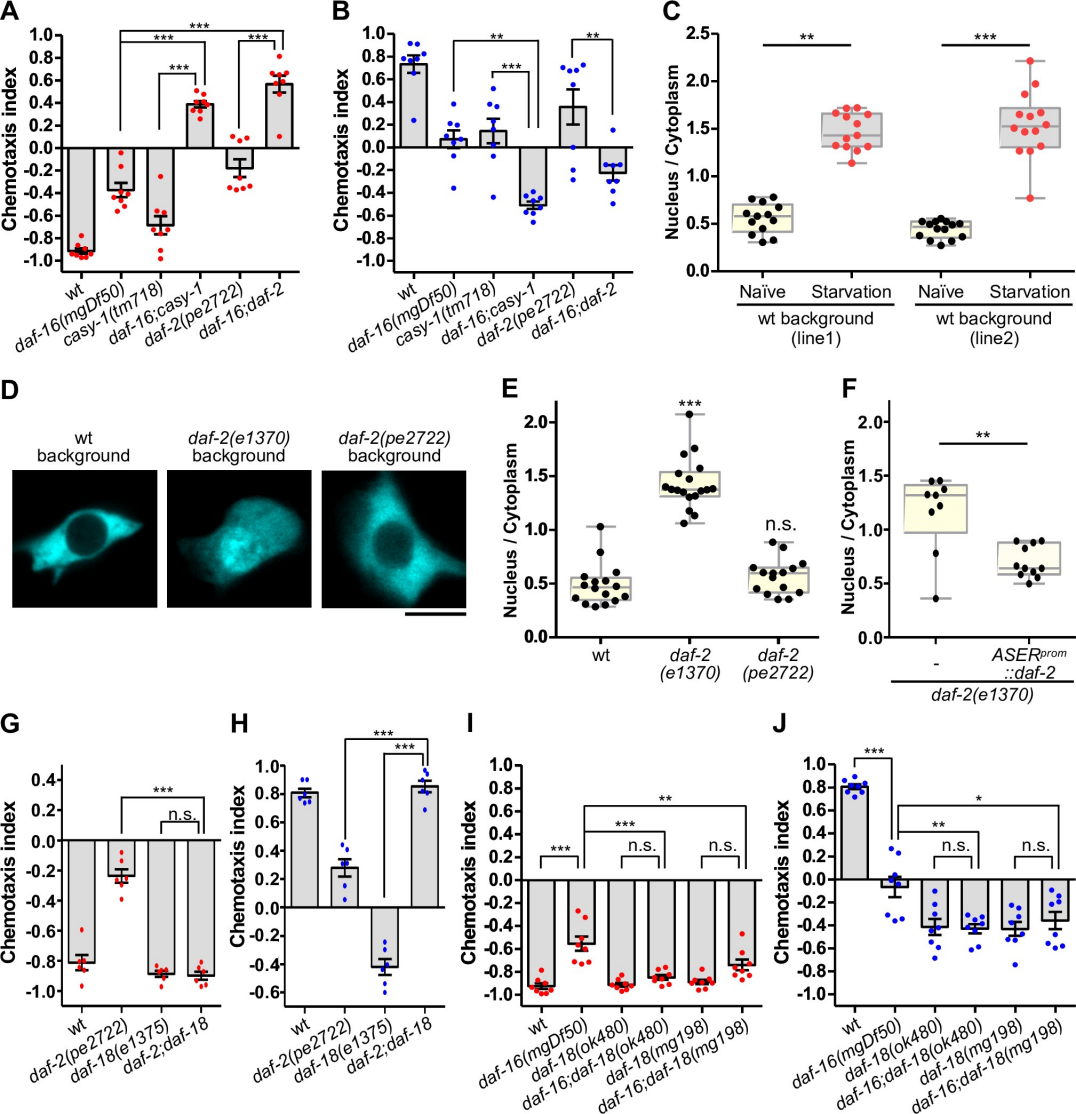
DAF-16 regulates taste avoidance learning in ASER independently of the DAF-2c pathway. (A, B) Salt chemotaxis after conditioning under starvation with high (A) or low (B) salt (N = 8). A red or blue dot represents a chemotaxis index obtained from each trial after conditioning with high or low salt, respectively. (C) Quantitative analyses of DAF-16::GFP localization in ASER before (naïve) and after starvation conditioning on agar plates containing 100 mM of salt for an hour (N > 12 animals). Fluorescence intensity ratios of the nucleus to the cytoplasm are shown. Two lines of animals, JN2837 and JN2885, were examined. (D, E, F) Analyses of DAF-16::GFP localization in ASER after incubation on an agar plate with 50 mM of salt and food at 25°C for two hours (E; N > 8 animals). Scale bar indicates 5 μm. (C, E) Dunn's multiple test following Kruskal-Wallis test, **p < 0.01 and ***p < 0.001. (F) DAF-2(exon 11.5-) was expressed by the ASER-specific *gcy-5* promoter. Mann Whitney test, **p < 0.01. (G-J) Salt chemotaxis after high- (G, I) or low-salt (H, J) conditioning in the absence of food. (A, B, G-J) Error bars indicate SEM. Tukey’s test following one-way ANOVA, n.s. p > 0.05, *p < 0.05, **p < 0.01, and ***p < 0.001.

We previously reported that insulin-like signaling acts in the axon of the ASER neuron in taste avoidance learning: During starvation conditioning, a splice isoform of the insulin receptor homolog, DAF-2c, is transported from the cell body to the axon of ASER, where it acts for taste avoidance learning. We looked into a possible interaction of DAF-16 with DAF-2c and CASY-1, the calsyntenin homolog required for translocation of DAF-2c to the axon. We generated the *daf-2c*-isoform specific mutant *daf-2c(pe2722)*, which harbors a frame-shift deletion in the *c* isoform-specific exon, and confirmed a substantial defect in taste avoidance learning, but not salt chemotaxis after feeding conditioning similar to a deletion mutant of *daf-16* or *casy-1* (Fig 3A and 3B, S3A and S3B Fig). The *daf-16(mgDf50)* mutation caused additive effects in the *daf-2c* and *casy-1* mutants. Furthermore, there was no substantial difference in the localization of DAF-2c::Venus in ASER between the wild-type and *daf-16* mutant animals; DAF-2c::Venus was observed throughout the whole ASER neuron, including the axon, both in the wild-type and the *daf-16* mutant (S3C Fig). These data suggest that DAF-16 functions in parallel with the CASY-1/DAF-2c pathway.

### DAF-16 translocation into the nucleus in ASER during starvation conditioning is dependent on insulin-like signaling

DAF-16 translocates into the nucleus during starvation where it controls the transcription of stress response genes [11]. *daf-16* mutants showed defects in learned behavior after starvation conditioning but was almost normal in salt chemotaxis after feeding conditioning, implying a role of DAF-16 in the transmission of the starvation signals during conditioning. Next, we investigated whether nuclear localization of DAF-16 was increased in the ASER neuron during starvation conditioning. DAF-16a::GFP was localized to the cytosol of ASER in well-fed animals, whereas it was translocated into the nucleus after starvation for an hour (Fig 3C). The DAF-16 nuclear translocation was not substantially different depending on salt concentrations during starvation (Fig 3C, S4A–S4C Fig), suggesting that the strong translocation occurs mainly due to starvation. The insulin receptor, DAF-2, a major upstream factor of DAF-16, negatively regulates nuclear localization of DAF-16 in several cell types [11,13]. In the background of the *daf-2(e1370)* reduction-of-function mutant, nuclear translocation of DAF-16a::GFP was significantly increased in the ASER neuron even under well-fed condition, and this phenotype was rescued by expression of DAF-2a in ASER (Fig 3D–3F). These results suggest that the DAF-2 insulin receptor acts upstream of DAF-16 cell-autonomously in ASER. On the other hand, the deletion mutation of *daf-2c*, *daf-2c(pe2722)*, had no significant effect on the localization of DAF-16a::GFP in ASER under well-fed conditions, suggesting that DAF-2 isoforms other than the *c* isoform likely repress DAF-16 nuclear translocation in ASER in the presence of food.

We previously reported that the PTEN phosphatase homolog DAF-18, a negative regulator of insulin-like signaling, functions in ASER in taste avoidance learning. *daf-18* mutants showed reduced high-salt migration irrespectively of the presence or absence of food during conditioning likely due to hyperactivation of the PI3K pathway [26]. The defect in high-salt migration of the *daf-18(e1375*rf*)* mutant was suppressed by the *daf-2c(pe2722)* mutation similar to the mutations of the PI3K homolog *age-1* (Fig 3H, S4D and S4E Fig; [26]). Conversely, the *daf-18(e1375*rf*)* mutation suppressed the taste avoidance learning defect of *daf-2c(pe2722)* (Fig 3G and 3H), suggesting that DAF-18 antagonizes the DAF-2c/PI3K pathway in taste avoidance learning. We next investigated the effect of a *daf-16* mutation on taste avoidance learning of *daf-18(ok480*rf*) and daf-18(mg198*null*)* mutants. If DAF-16 is negatively regulated by insulin-like signaling in taste avoidance learning, it is expected that the effect of the *daf-16* mutation on the learning is reduced in the *daf-18* mutants compared to the wild type, because in the *daf-18* mutants the activity of DAF-16 will be already reduced in the *daf-16(+)* background. As we expected, the *daf-16* mutation had no significant effect on taste avoidance learning in the background of the *daf-18* mutants (Fig 3I and 3J). On the other hand, the *daf-18* mutations significantly altered salt chemotaxis of the *daf-16* mutant, consistent with the notion that DAF-18 modulates both the axonal DAF-2c signaling and the cell boy DAF-2/DAF-16 pathway in parallel. These data support the view that insulin-like signaling mediated by DAF-2 isoforms other than DAF-2c negatively regulates DAF-16 functions in taste avoidance learning.

### Forced nuclear localization of DAF-16 promotes low-salt migration

**Fig 4:**
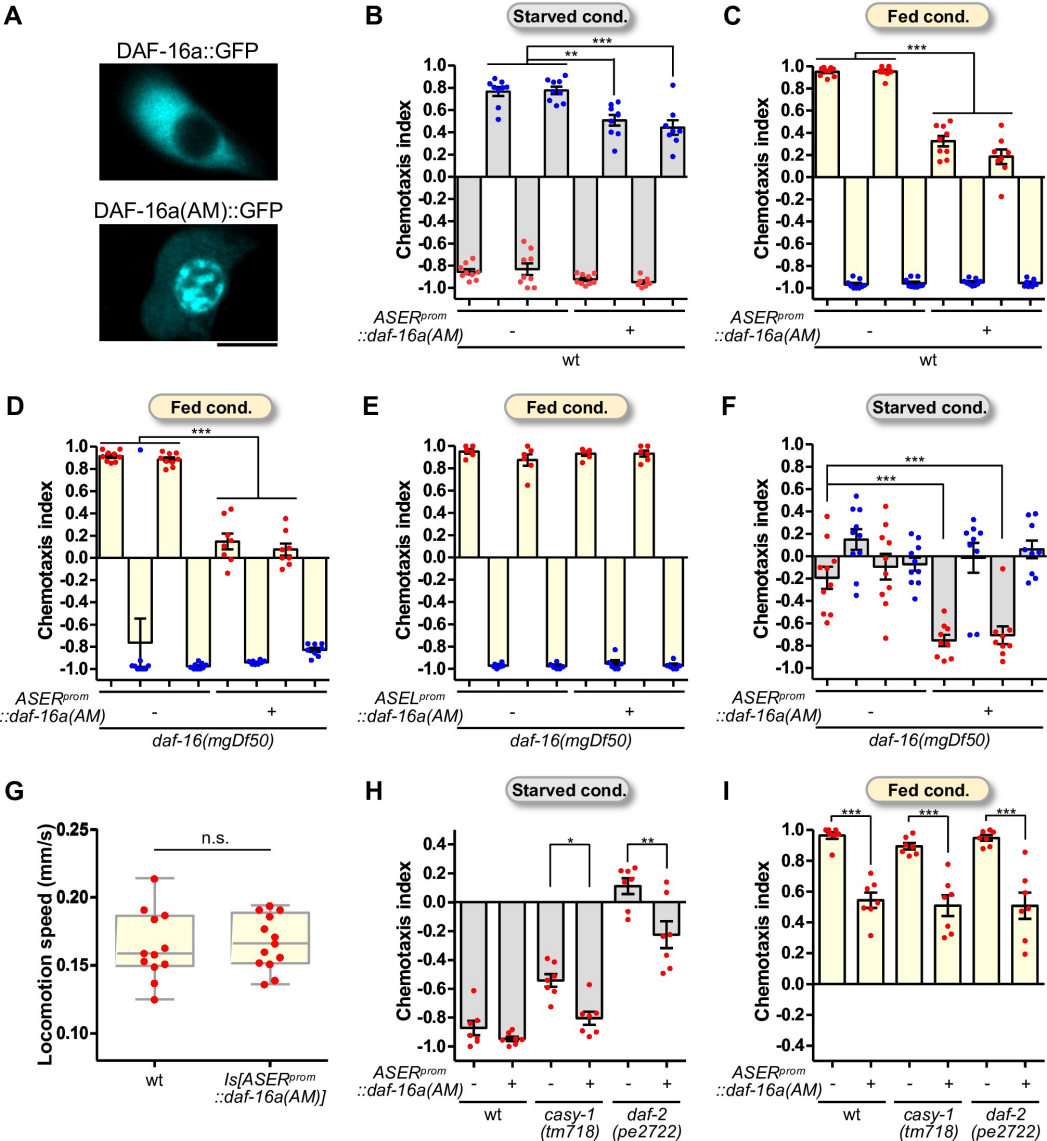
Forced nuclear localization of DAF-16 in ASER biases chemotaxis towards low salt. (A) Representative images of subcellular localization of GFP-fused DAF-16a and DAF-16a(AM) in ASER. Scale bar indicates 5 μm. (B, C) The effect of DAF-16a(AM) expression in ASER in salt chemotaxis after starvation conditioning (B) or feeding conditioning (C) in the wild-type background (N = 8 or 9). A red or blue dot represents a chemotaxis index obtained from each trial after conditioning with high or low salt, respectively. Two lines of transgenic animals were used. (D, E, F) The effect of DAF-16a(AM) in the *daf-16* mutant background (N > 5). Chemotaxis indices after feeding conditioning (D, E) or starvation (F) are shown. DAF-16a(AM) was expressed in ASER (D, F) or ASEL (E) under the *gcy-5* or *gcy-7* promoter, respectively. (G) Locomotor speed of the wild type and the JN2874 strain (*Is[gcy-5p*::*daf-16a(AM)]*) (N > 11 trials). A red dot represents an average value of locomotion speed of animals in each trial. Mann Whitney test, n.s.p > 0.05. (H, I) The effect of DAF-16a(AM) expression in ASER on salt chemotaxis after high-salt conditioning without (H) or with (I) food in the wild type and *casy-1* and *daf-2* mutants (N = 7). Error bars indicate SEM. Tukey’s test following one-way ANOVA, * p < 0.05, **p < 0.01, and ***p < 0.001.

It has been reported that DAF-16 activity is negatively regulated by its phosphorylation via AKT-1 and AKT-2 kinases, and a mutant form of DAF-16, in which serine/threonine residues in the putative AKT-1-phosphorylation sites were substituted by alanine, was strongly localized to the nucleus [13,29]. We expressed the mutant form of DAF-16a (hereafter, DAF-16a(AM)) in the ASER neuron, and confirmed that DAF-16a(AM)::GFP was strongly localized to the nucleus in ASER even after well-fed conditions (Fig 4A, S5A and S5B Fig). We next examined the effect of DAF-16a(AM) expression in ASER or ASEL on behavior. The wild-type or *daf-16* mutant animals expressing DAF-16a(AM) in ASER showed significant decreases in high-salt migration after low-salt/starvation and/or high-salt/feeding conditioning (Fig 4B, 4C and 4D). Meanwhile, the effect of DAF-16a(AM) on salt chemotaxis was not observed when expressed in ASEL (Fig 4E). Combined with the finding that ASER expression of DAF-16 or DAF-16a(AM) was sufficient for rescue of the learning defect in the *daf-16* mutant (Fig 2B, Fig 4F), these results suggest that the action of DAF-16 in the nucleus of ASER drives low-salt migration after starvation conditioning with high salt. On the other hand, no substantial rescue was observed by ASER expression of DAF-16a(AM) in the high-salt migration defect of the *daf-16* mutant (Fig 4F), suggesting that an appropriate level of DAF-16 activity is required for high-salt migration after starvation conditioning. The chemotaxis defect toward high salt observed by the expression of DAF-16a(AM) was not due to locomotory defect at high salt (Fig 4G). The DAF-16a(AM) expression increased low-salt migration also in the *casy-1* and *daf-2c* mutants, similar to that in the wild type (Fig 4H and 4I), suggesting that DAF-16 nuclear localization biases chemotaxis toward lower salt concentrations independently of axonal insulin-like signaling in ASER.

### Neuropeptide signaling is required for DAF-16-dependent low-salt migration

**Fig 5:**
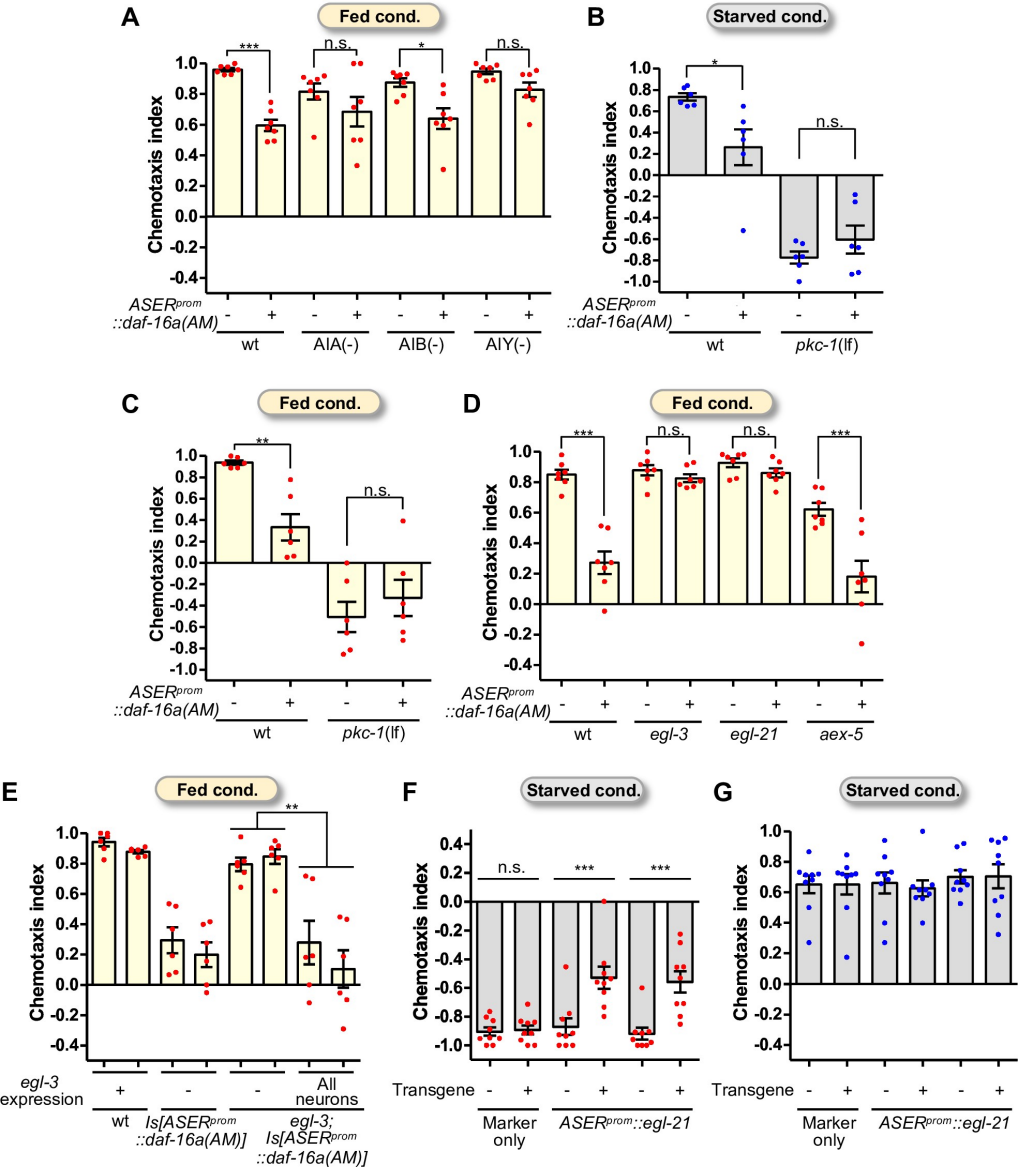
Neuropeptide signaling is required for DAF-16-dependent salt chemotaxis plasticity. (A) The effect of DAF-16a(AM) on salt chemotaxis after high-salt/feeding conditioning in interneuron-ablated animals (N = 7). (B, C) The effect of DAF-16a(AM) on salt chemotaxis after low-salt/starvation (B) or high-salt/feeding (C) conditioning in *pkc-1* loss-of-function mutants (N = 6). (D, E) Mutants of neuropeptide-processing enzymes (D; N = 7) and transgenic *egl-3* mutant animals (E; N = 6). *egl-3* cDNA was expressed by the pan-neuronal *H20* promoter. (F, G) The effect of *egl-21* expression in ASER on salt chemotaxis after high- or low-salt/starvation conditioning (N = 9). Two lines of *egl-21* transgenic animals were used. Each dot in red (A, C, D-F) and blue (B, G) represents a chemotaxis index obtained from each trial after conditioning with high or low salt, respectively. Error bars indicate SEM. Tukey’s test following one-way ANOVA, n.s. p > 0.05, *p < 0.05, **p < 0.01, and ***p < 0.001.

We sought to uncover the molecular mechanisms underlying the DAF-16-dependent salt chemotaxis plasticity. We first examined the interneurons required for DAF-16-dependent low-salt migration. DAF-16a(AM) was expressed in animals in which the postsynaptic interneurons of ASER, either AIA, AIB, or AIY, were ablated by mouse caspase expression [34], and examined the effect on salt chemotaxis of those animals. The effect of DAF-16a(AM) expression was decreased in AIA- or AIY-ablated animals, as there was no significant change in high-salt migration after feeding conditioning by DAF-16a(AM) expression (Fig 5A). These results imply that ASER transmits DAF-16-dependent signals to AIA and AIY interneurons to drive migration to the low-salt area. We next examined the possible role of DAF-16 in neurotransmission. Diacylglycerol (DAG) signaling regulates neurotransmission through the nPKC-ε/η ortholog, PKC-1 (S5C Fig) [35,36], and plays a key role in salt chemotaxis plasticity in ASER [37]. *goa-1*, encoding a Goα subunit, and *egl-30*, encoding a Gqα subunit, have been proposed to regulate salt chemotaxis mainly through DAG signaling in ASER (S5C Fig). Both loss-of-function (lf) of *goa-1* and gain-of-function (gf) of *egl-30* increase high-salt migration, whereas a lf mutation of *pkc-1* increases low-salt migration [25,26,37]. Increased low-salt migration by DAF-16a(AM) was suppressed by *goa-1(n1134*lf*)* or *egl-30(pe914*gf*)* mutations (S5D Fig), suggesting that the DAG/PKC-1 pathway acts downstream of or in parallel with DAF-16a(AM). The effect of DAF-16a(AM) was not observed in the background of a null mutant of *pkc-1*, *pkc-1(nj3*lf*)* (Fig 5B and 5C). These data suggest that the DAG/PKC-1 pathway mediates DAF-16-dependent low-salt migration.

The DAG/PKC-1-dependent pathway is involved in neurotransmission via neuropeptides [36]. In *C*. *elegans*, precursor proteins of neuropeptides are cleaved by proprotein convertases which are encoded by four genes, namely PC1/3 homologs, *kpc-1*, *bli-4*, and *aex-5*, and the PC2 homolog *egl-3*. The C-terminal extensions of lysine and/or arginine residues of these cleaved peptides are subsequently removed by carboxypeptidases E, such as EGL-21. Mutants of these enzymes actually cause changes in the production of many neuropeptides [38,39]. As mutants of *aex-5* and *egl-3* showed significant defects in salt chemotaxis plasticity (S6A–S6D Fig), we examined the effect of DAF-16a(AM) expression in the *aex-5* and *egl-3* mutants and found that ASER expression of DAF-16a(AM) significantly reduced high-salt migration of the *aex-5* mutant, but not the *egl-3* mutant (Fig 5D), suggesting that decreased high-salt migration caused by DAF-16a(AM) expression requires the EGL-3 neuropeptide processing enzyme. We confirmed that *egl-3* expression in the whole nervous system was sufficient for the rescue of the effect of DAF-16a(AM) expression and the salt chemotaxis defect in the *egl-3* mutant (Fig 5E, S6E and S6F Fig). We note that DAF-16a(AM) expression in ASER in some mutants caused salt chemotaxis defects in a manner different from that in the wild type: it significantly promoted migration to higher salt concentrations in *egl-3*, *egl-21* and *goa-1* mutants and AIB-ablation animals (S7 Fig; see also Discussion).

Most neuropeptides processed by EGL-3 are thought to be further processed by EGL-21 [39]. Indeed, DAF-16a(AM) expression had no significant effect also in an *egl-21* mutant background (Fig 5D). Hence, we next examined the expression levels of *egl-21*, *egl-3*, and some other genes related to neurotransmission by qRT-PCR. No significant change was observed in expression of *egl-3*, *pkc-1*, *unc-13*, and *eat-4*, which encodes the vesicular glutamate transporter, by DAF-16a(AM) expression in ASER. On the other hand, the DAF-16a(AM) expression weakly, but significantly, reduced expression of *egl-21* and *dgk-1*, the latter of which encodes a diacylglycerol kinase (S8B Fig). Moreover, overexpression of *egl-21* in ASER imposed migration bias to higher salt concentrations after high-salt/starvation conditioning, whereas it had no significant effect on chemotaxis after low-salt/starvation conditioning (Fig 5F and 5G), a phenotype opposite to that caused by DAF-16a(AM) expression in ASER. These results are consistent with the notion that DAF-16 promotes low-salt migration by changing production of neuropeptides via the EGL-21 neuropeptide processing enzyme in ASER.

## Discussion

### Role of DAF-16/FOXO in taste avoidance learning

**Fig 6:**
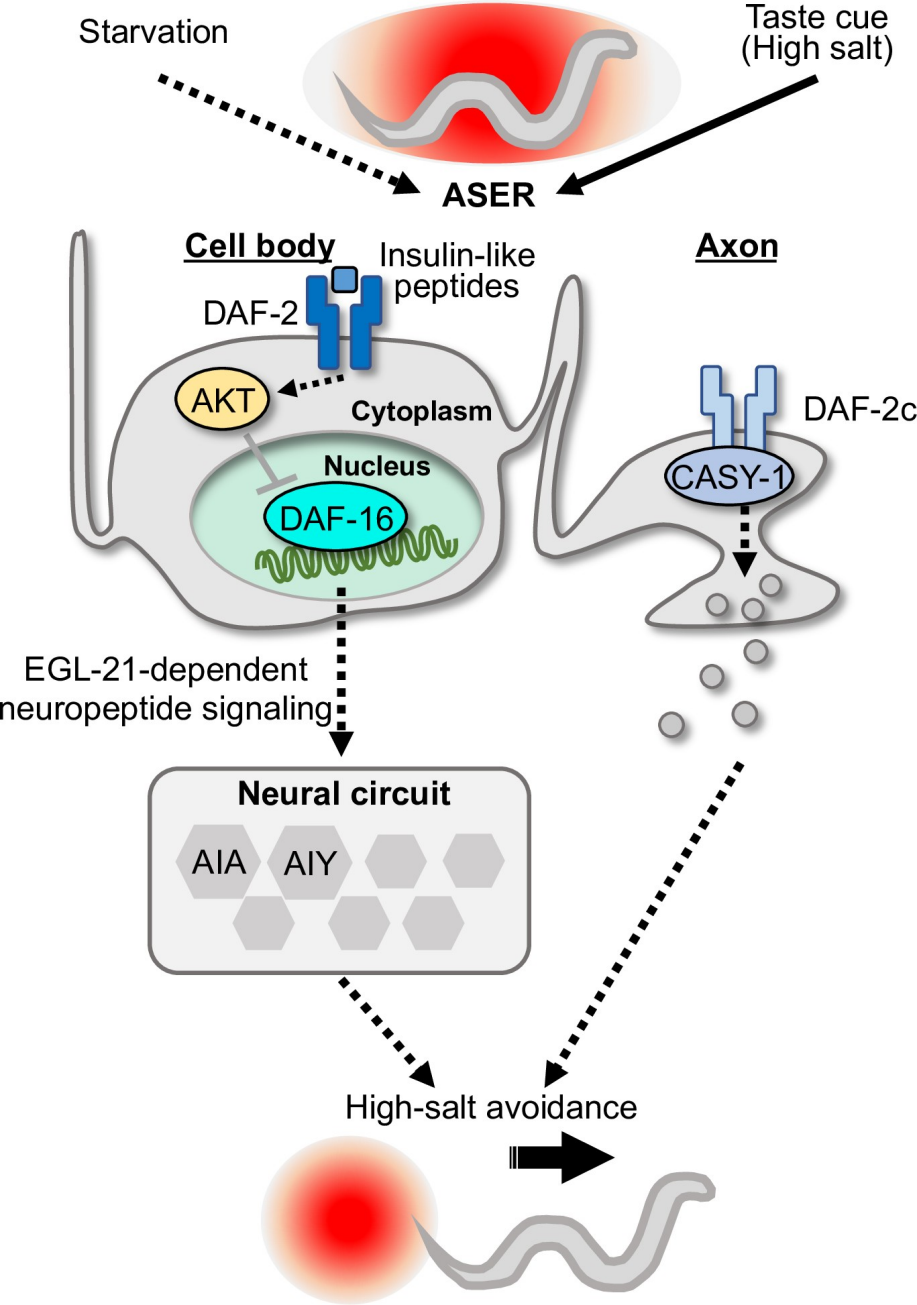
A summary of DAF-16 functions in taste avoidance learning. Animals avoid salt concentrations encountered during starvation and migrate to areas at lower and higher salt concentrations. To avoid high-salt areas, DAF-16 acts in the ASER cell body independently of axonal DAF-2c signaling. DAF-16 is translocated into the nucleus of ASER dependent on signaling mediated by DAF-2(exon 11.5-) isoforms and transmits the signal to downstream neurons, including AIA and AIY, via neuropeptide signaling modulated by EGL-21/carboxypeptidase E, and promotes high-salt avoidance.

Taste avoidance learning is a form of associative learning between starvation conditions and salt concentrations. We previously reported that the Ras/MAPK signaling pathway mediates transmission of food signaling to the ASER neuron and its downregulation increases axonal transport of DAF-2c, by which salt avoidance is promoted [25]. In this study, we show that DAF-16 acts in parallel to the DAF-2c pathway in taste avoidance learning. DAF-16 was translocated into the nucleus of ASER under fasting conditions in a DAF-2-dependent manner, and this regulation was mediated by DAF-2(exon 11.5-) isoforms (see below). Constitutively nuclear-translocated DAF-16, DAF-16a(AM), increased salt avoidance even after feeding conditioning. Furthermore, a cell type- and timing-dependent expression using the auxin-inducible degradation system revealed that DAF-16 acts in ASER around the time of taste avoidance learning. These observations suggest that DAF-2(exon 11.5-)/DAF-16 signaling likely transmits starvation signals to the ASER nucleus in parallel to the axonal DAF-2c pathway in taste avoidance learning. This dual function of insulin-like signaling in the cell body and the axon may ensure dynamic changes of behavioral responses after starvation conditioning (Fig 6).

*daf-16* mutations caused strong defects in learned behaviors after conditioning under starvation (Fig 1, S3A Fig), but only minor effects on those after feeding conditions, suggesting that the defects of the *daf-16* mutants are not simply due to abnormalities in salt sensitivity or locomotory activity. The effect of DAF-16a(AM) expression was significantly decreased in animals with genetically ablated AIA and AIY interneurons. These findings imply that DAF-16 may regulate salt avoidance by controlling neurotransmission between ASER and the innervated interneurons, such as AIA and AIY. It was reported that the morphology of AIY is regulated by DAF-16, especially a DAF-16b isoform, during development [31]. Therefore, loss of the DAF-16b isoform might cause a change in neurotransmission from ASER to AIY, possibly leading to defective learning behavior. However, the *b* isoform-specific *daf-16* mutant had no significant defect in the learning (Fig 2D and 2E). In addition, not only DAF-16b but also DAF-16a and DAF-16f rescue the defect in the learning (Fig 2B), while expression of DAF-16a and DAF-16f did not rescue abnormal morphology of AIY [31]. Therefore, it seems that the learning defects of the *daf-16* mutants are not caused only by abnormal morphology of AIY. The AIY interneurons mediate an ASER-dependent curved locomotion toward low-salt concentrations [34]. The AIA and AIY interneurons are activated upon an increase in salt concentration and promote forward locomotion [40]. It will be interesting to investigate how DAF-16 controls the activities of interneurons upon ASER activation and the regulation of taste avoidance learning.

### Evolutionarily conserved roles of insulin/FOXO signaling in feeding-related behavior

Nuclear localization of DAF-16 was increased in ASER in *daf-2(e1370)*, a reduction-of-function mutation in the exon common to all isoforms, but not in *daf-2(pe2722)*, a frame-shift-deletion mutation in exon 11.5 which is contained only in the *daf-2*c isoform, suggesting that DAF-16 is negatively regulated by exon 11.5-skipped DAF-2, DAF-2(exon 11.5-), which preferentially localizes to the cell body, as compared to DAF-2c(exon 11.5+) (Fig 3E) [28]. The observation that DAF-16 nuclear localization is dependent on the putative Akt phosphorylation sites (Fig 4A) suggests that the cell body isoform, DAF-2(exon 11.5-), regulates DAF-16 localization via Akt in ASER, similar to that in other cell types [11,13,29]. Also, in mammalian neurons, the receptors for insulin-like peptides, such as insulin, IGF-I, and IGF-II, are localized to both the cell body and axon [41–43]. However, the relationship between multiple insulin-like signaling in the cell body and axon within a single neuron remains unclear. In *C*. *elegans*, impairment of both nuclear and axonal insulin-like signaling caused salt attraction after starvation conditioning as if the mutant worms were conditioned in the presence of food (Fig 3A and 3B). Therefore, the cooperative function of insulin-like signaling acting in the distinct subcellular regions is essential for generation of the behavioral mode of salt avoidance caused by starvation conditioning, although the precise timing of action of these signaling pathways are currently unknown.

In axonal DAF-2 signaling, the insulin-like peptide INS-1, which preferentially localized to the axonal processes, regulates taste avoidance learning [25,26]. On the other hand, for the DAF-2(exon 11.5-)/DAF-16 signaling pathway in the cell body, an insulin-like peptide that regulates taste avoidance learning remains unclear. Because decision of dauer entry and longevity are modulated by the presence or absence of food, the activity of DAF-2(exon 11.5-)/DAF-16 signaling in ASER might be regulated by starvation responses commonly used for the regulation of development and longevity. In mammals, insulin released from the peripheral tissues enters the brain and acts on the insulin receptors expressed in the hypothalamic arcuate nucleus and regulates food intake behavior through control of FOXO1-dependent expression of neuropeptides, such as agouti-related protein (AgRP) and pro-opiomelanocortin (POMC) [44]. In this study, DAF-16 was found to regulate salt chemotaxis through EGL-3- and EGL-21-dependent neuropeptide processing in the nervous system. Taste avoidance learning of *C*. *elegans* is believed to increase the chance of obtaining food by avoiding areas that are likely devoid of food. Therefore, the insulin/FOXO pathway-dependent modulation of neuropeptide signaling might be an evolutionarily conserved mechanism to control feeding-related behavior.

### Molecular mechanisms underlying FOXO-dependent regulation of learning and memory

Although DAF-16 mainly localizes to the cytoplasm in the presence of food, the *daf-16(mgDf50*null*)* mutant exhibited a mild defect in high-salt migration after feeding conditioning (S3A Fig, S4 Fig). Thus, a weak activity of DAF-16 in the nucleus is likely required for high-salt attraction after feeding conditioning. On the other hand, starvation strongly promotes nuclear localization of DAF-16 independent of salt concentrations during fasting (Fig 3C, S4 Fig). The *daf-16(lf)* mutants showed strong defects in migration to both low- and high-salt concentrations after starvation conditioning (Fig 1). Thus, DAF-16 is required for avoidance of the salt concentrations associated with starvation towards both lower and higher concentrations. We speculate that DAF-16 controls transcription of genes required for the behavioral switch caused by starvation conditioning and those genes promote both low- and high-salt migration dependent on the cellular environments after high- and low-salt conditioning, respectively. The forced nuclear localization by DAF-16a(AM) expression promotes low-salt migration irrespectively of the presence or absence of food during conditioning (Fig 4B and 4C). The forced nuclear-localized DAF-16 may affect transcription of only a subset of genes that regulate salt chemotaxis, such as *egl-21* and *dgk-1*, thereby causing unbalanced promotion of lower salt migration or repression of higher salt migration. Interestingly, in worms with defective neuropeptide processing, DAG metabolism, or AIB function, the forced DAF-16 nuclear localization in ASER significantly promoted migration to higher salt (S7 Fig). These observations support the notion that DAF-16 can promote salt chemotaxis in different directions dependent on the cellular environments of the neural circuits. Because DAF-16a can also function in muscle cells and chemosensory neurons other than ASER in taste avoidance learning, in addition to the cell-autonomous functions in ASER, DAF-16a might regulate taste avoidance learning from neuronal and muscle cells to the taste neural circuit in a cell-non-autonomous manner. Detailed mechanisms underlying several modes of actions of DAF-16 in taste avoidance learning will need to be further investigated.

A previous study demonstrated that a mutation in carboxypeptidase E was associated with the development of neurodegenerative diseases [45], suggesting that processing of neuropeptides plays a key role in neural function. FoxO1 negatively regulates the expression of carboxypeptidase E in POMC-expressing neurons in mice, which leads to reduced food intake through processing of POMC [46]. Our qRT-PCR and behavioral analyses suggest that DAF-16/FOXO also negatively regulates the expression levels of EGL-21/carboxypeptidase E (S8B Fig), thereby promoting avoidance of salt concentrations associated with starvation. EGL-21 overexpression in ASER caused the taste avoidance learning defect (Fig 5F) in the same direction as the *egl-21(lf)* mutant (S6A Fig), consistent with the notion that an adequate level of EGL-21 in ASER is required for taste avoidance learning. FOXO-dependent neuropeptide processing may underlie feeding-related behavior and behavioral plasticity across species. As the recovery of neuropeptide production by *egl-3* expression in the whole nervous system was sufficient for rescue of the DAF-16-induced salt avoidance deficiency in the EGL-3/PC2 mutant, DAF-16 appears to regulate taste avoidance learning via neuropeptide signaling acting in unknown neuron(s) in the taste neural circuit (Fig 6). In addition to *egl-21*, expression of a diacylglycerol kinase *dgk-1* was significantly reduced in the DAF-16a(AM)-expressing animals (S8F Fig). Mutations of *dgk-1*, which are predicted to increase DAG levels, were reported to cause defects in associative learning between odor and starvation and suppress decreased odor chemotaxis in *daf-18* mutants similar to those of the insulin-like pathway [47,48]. The axonal DAF-2 signaling regulates DAG dynamics in response to salt concentration changes [27]. In this study, we show that the DAF-16-dependent low-salt migration requires normal DAG/PKC-1 signaling (Fig 5B and 5C, S5D Fig). These findings imply that DAG/PKC-1 signaling may play a key role downstream of both the soma and axonal DAF-2 pathways. Further comprehensive analysis will be required to understand fully the DAF-16-dependent transcriptional regulation acting during taste avoidance learning. Based on our study, we propose a speculative model for the regulatory functions of DAF-16 in taste avoidance learning (Fig 6).

The insulin-FOXO axis is likely to be important for cognitive functions in mammals, including humans. Insulin receptors are expressed in the cerebral cortex and the hippocampus, which play critical roles in learning and memory [49]. FOXO1, FOXO3, and FOXO6 are expressed in the murine brain [50]. Cyclin-dependent kinase-5 (Cdk5) increases amyloid beta levels through FOXO3 activity and induces the pathogenesis of Alzheimer’s disease [51]. FOXO6 expressed in the hippocampus is required for memory consolidation and synaptic function [52]. We believe that our findings will promote the discovery of novel functions of insulin/FOXO signaling in the complex nervous system.

## Methods

### *Caenorhabditis elegans* strains

The list of *C*. *elegans* strains is shown in S1 Table. The Bristol N2 strain was used as wild type. The *C*. *elegans* strains were cultured as described [53]. Animals were cultivated on Nematode Growth Media (NGM) plates under 15°C, 20°C, or 25°C. An *Escherichia coli* strain, NA22, was used as a bacterial diet. Double mutants were generated by genetic crossing, and their genotypes were checked by PCR.

### DNA constructs and transgenesis

For the *daf-16* rescue experiments, the cDNA and a promoter region of each *daf-16* isoform were amplified with KOD-Plus-Neo DNA polymerase (Toyobo, Japan). A promoter region of each *daf-16* isoform was amplified with KOD-Plus Neo DNA polymerase using wild-type (N2) total cDNA and genomic DNA as a template, respectively. Primers are shown in S2 Table. The 6.1 kbp, 4.0 kbp, and 3.4 kbp 5’ upstream regions were used for the promoter regions of *daf-16a*, *daf-16b*, and *daf-16f*, respectively, as referred in Kwon *et al*., (2010). Most of the *daf-16* expression plasmids were generated by the Gateway system (Thermo Fisher Scientific). The PCR-amplified *daf-16* cDNAs were inserted into the NheI-KpnI site of the pDEST vector containing an *sl2*::*venus* sequence (pDEST-*sl2*::*venus*). The PCR-amplified *daf-16* promoter sequences were inserted into the BamHI-NotI site of the pENTR vector (pENTR-1A). The promoter region was inserted upstream of each *daf-16* isoform cDNA by the LR reaction using the LR reaction kit (Thermo Fisher Scientific, Japan). Plasmid DNA was transformed into *E*. *coli* competent cells (DH5α or DB3.1) and extracted by the GENE-PREP-SYSTEM (KURABO, Japan). Plasmids were purified by using the QIAquick PCR Purification Kit (QIAGEN, Japan). The expression constructs for *daf-16* were injected into animals at 5 ng μl^-1^ with a *myo-3*^*prom*^::*venus* transformation marker (10 ng μl^-1^) and an empty vector (pPD49.26).

A *daf-16a(AM)*::*gfp*-expressing plasmid was generated by PCR-based mutagenesis of the pGP30 plasmid including *daf-16*^*prom*^::*daf-16a*::*gfp* [13]. Both serine and threonine codons in the four putative Akt phosphorylation sites (T54, S238, T240, and S312) were mutated to an alanine codon (GCT). Transgenic animals carrying *daf-16a(AM)*::*gfp* transgenes were generated by introduction of the *daf-16a(AM)*::*gfp*-expressing plasmid (5 ng μl^-1^) with a *myo-3*^*prom*^::*venus* transformation marker (10 ng μl^-1^) and an empty vector (pPD49.26).

*egl-21* cDNA was amplified from N2 total cDNA with KOD-One (Toyobo) DNA polymerase using primers, 5’-AGTAGCTAGCATGCTGCACGCGATGCG-3’ and 5’-GGACGATATCTTAACGACGACGGGCAATC-3’.

A *daf-2*(*pe2722)* mutant was generated by the CRISPR/Cas9 system. A Cas9 target site, 5'-GACGATGAAGAGCCCGGCGG-3', was inserted into the pDD162 (*eft-3*^*prom*^::*Cas9* + *Empty sgRNA*) vector as previously described [54]. The pDD162 vector containing the Cas9 target site was injected into animals at 50 ng μl^-1^ with a *myo-3*^*prom*^::*venus* transformation marker (10 ng μl^-1^) and an empty vector, pPD49.26 (40 ng μl^-1^). The progenies with short deletions in exon 11.5 of the *daf-2* gene were selected by PCR. The *pe2722* mutation contains 41 bp deletion in exon 11.5.

### Salt concentration learning test

To examine salt concentration learning, we performed salt chemotaxis assays after conditioning on agar plates with several different sodium chloride (NaCl) concentrations in the presence or absence of food. Salt chemotaxis assays were performed essentially as previously described [24,28]. Animals were grown to the adult stage on NGM plates seeded with *E*. *coli* NA22 at 20C. For conditioning, adult animals were collected with wash buffer (50 mM NaCl, 25 mM KPO_4_, 1 mM MgSO_4_ 1 mM CaCl_2_, and 0.2% gelatin), washed twice and then transferred to agar plates containing 25 or 100 mM NaCl with or without NA22. Animals were conditioned for six hours at 20C. The animals conditioned with food were collected with wash buffer, washed twice and placed at the center of a test plate. The animals conditioned without food were collected with wash buffer and transferred to a test plate. Animals were allowed to move freely for 45 minutes on the test plates. To prepare the test plate, two agar blocks (14 mm in diameter, including either 150 mM NaCl (higher side) or 0 mM (lower side)) were placed at the positions 3 cm away from the center of a 9 cm agar plate 18–24 hours before behavioral tests. The agar blocks were removed and one microliter of 0.5 M sodium azide was spotted at those positions just before behavioral tests to immobilize animals around the spots. After behavioral tests, the test plates were transferred to 4°C refrigerator in order to stop the movements of all animals on the plates. The number of animals was counted in each region of test plates, and then the chemotaxis index was calculated by the equation as follows, where N_high-salt_ is the number of animals in the high-salt region, N_low-salt_ is that in the low-salt region, N_start point_ is that in the starting region and N_all_ is the number of all animals on test plates. Schematic of test plates was shown in our previous paper [28]. In many cases, we used 50–200 animals for each chemotaxis test.

Chemotaxis index = (N_high-salt_ - N_low-salt_) / (N_all_ - N_start point_)

In an auxin-inducible degradation assay, both *degron*::*daf-16a*, *daf-16a* cDNA N-terminally tagged with the *degron* sequence, and *TIR1* cDNA were expressed in the ASER neuron. Sequences of *degron* and the *TIR1* gene were shown [33]. Auxin (Alfa Aesar (#A10556)) was dissolved in ethanol at 4.0 x 10^2^ mM to prepare an auxin stock solution. Day 1 adult animals were transferred to NGM plates seeded with NA22, which contain 1 mM auxin or 0.25% ethanol as a control. After incubation for two hours at 20°C, animals were collected with wash buffer, washed twice, and then transferred to a conditioning plate, which contains 100 mM NaCl and 1 mM auxin or ethanol (control) without food. Chemotaxis of the conditioned animals was tested as described above.

### Observation of fluorescence gene expressions

Animals were grown to the adult stage at 20°C. Day 1 adult animals were mounted and anaesthetized on a 5% agar pad containing 10 mM sodium azide. In addition to bright field images, fluorescence images of Venus, mCherry and tagRFP were acquired by using a confocal microscopy (Leica SP5) with 514, 543, and 633 nm excitation laser lights, respectively, and a 63 × objective.

### Quantitative analysis of DAF-16::GFP subcellular localization

Animals at the L4 stage were transferred to a fresh NGM plate seeded with *E*. *coli* NA22, which contains 50 mM of NaCl. After 24–30 hours, animals were mounted and anaesthetized on a 5% agar pad containing 10 mM sodium azide. In Fig 3D–3F, adult animals were mounted on the agar pad after further incubation for two hours at 25°C. Images were taken within 10 to 60 minutes after animals were mounted. In addition to bright field images, fluorescence images of GFP, mCherry, and tagRFP were acquired by using a confocal microscopy (Leica SP5) with 488, 543, and 633 nm excitation laser lights, respectively, and a 63 × objective. Z-stack images were taken with 0.2 μm spacing. DAF-16::GFP fluorescence intensity ratios of the nucleus to the cytoplasm were measured by ImageJ Fiji (version 1.0). An image containing the largest area of the ASER cell body was selected from z-series images based on tagRFP expression. A region of the ASER nucleus was manually determined in the selected image based on mCherry expression. An average fluorescence intensity of DAF-16::GFP was calculated in each region of the nucleus and the cytoplasm (that is, the peripheral region of the nucleus), and then the intensity ratio of the Nucleus/Cytoplasm was calculated.

### Measuring locomotor speed

Animals were grown to the adult stage on NGM plates seeded with NA22 at 20°C and conditioned on a plate with 100 mM NaCl in the presence of food for 5–7 hours at 20°C. They were washed twice and transferred to a test plate containing 100 mM NaCl. Images of worms on the test plate were acquired for 15 minutes at a frame per second, and then locomotor speeds of the animals were calculated based on trajectories of the centroids of the animals using the worm tracking system as previously reported [24].

### Real-time PCR analysis

Total RNA was extracted from wild-type animals and two lines of transgenic animals expressing DAF-16a(AM) in ASER (JN2874 and JN3212 strains). cDNA was synthesized by using PrimeScript RT reagent Kit (Takara). The cDNA was used as templates for real-time PCR analysis using SYBR PreMix ExTaqII (Takara). The list of primers is shown in S3 Table. The analyses were performed using Thermal Cycler Dice Real Time System (Takara). The parameters for PCR were as follows: 95°C incubation for 30 seconds followed by 40 cycles of 95°C incubation for 5 seconds and 60°C incubation for 30 seconds. The expression level was normalized to that in the wild type and an *eft-3* gene was used as an internal standard.

### Statistical analysis

One-way ANOVA with Tukey’s and Dunnett’s multiple comparison tests were used for statistical analyses. Two-way ANOVA with Bonferroni’s multiple comparison was used for statistical analyses for auxin-inducible degradation assays. Kruskal-Wallis and Mann Whitney tests were used for statistical analyses of real-time PCR assays and quantification of DAF-16::GFP localization. These analyses were performed by using GraphPad Prism 5.0 software (GraphPad Software, La Jolla, CA).

## Supporting information

S1 Fig Expression of each DAF-16 isoform. (A) The genomic structures of *daf-16* isoforms, and the promoter regions used for expression and behavioral analyses are shown. (B-D) Expression patterns of Venus driven by the promoters of *daf-16a*, *daf-16b*, and *daf-16f*. Venus and each DAF-16 isoform are co-expressed from the *venus*-fused *daf-16* isoform pre-mRNA separated by a sequence with an acceptor site of a spliced leader (SL2) *trans*-splicing, *daf-16*::*sl2*::*venus*. I, intestine; M, muscle; P, pharynx; S, spermatheca. Scale bars indicate 0.2 mm. (E-G) Expression patterns of the a, b, and f isoforms in the head region. N, neurons; P, pharynx; I, intestine; M, body wall muscle. Scale bar indicates 30 μm. (H-J) ASER expressions of DAF-16 isoforms were confirmed by co-expression of tagRFP driven by the ASER-specific *gcy-5* promoter. Scale bar indicates 30 μm.
(PDF)

S2 Fig Rescue experiments of taste avoidance learning defects in *daf-16* mutants. (A-D) Salt chemotaxis after high- (A, D) or low-salt (B, C) conditioning without food in *daf-16* transgenic animals. A red or blue dot represents a chemotaxis index obtained from each trial after conditioning with high or low salt, respectively. A repeat of the experiment shown in Fig 2B (A; N = 7). Multiple *daf-16* isoform cDNAs were expressed in *daf-16(mgDf50)* mutant animals by the endogenous promoters or the ASER-specific *gcy-5* promoter (B; N = 7 or 8). A DAF-16a isoform was expressed in all or most neurons, amphid and phasmid sensory neurons except ASE and AFD, the intestine, the muscle and the hypodermis of *daf-16* mutant animals by the *H20*, *odr-4*, *ges-1*, *myo-3*, and *dpy-7* promoters, respectively (C; N = 8). (C, D) Salt chemotaxis after high-salt conditioning without food in *daf-16* transgenic animals. *daf-16a*::*gfp* was expressed in *daf-16* mutant animals under the endogenous promoter, the ASER-specific *gcy-5* promoter or the ASEL-specific *gcy-7* promoter (D; N = 9). Error bars indicate SEM. Tukey’s test (A) or Dunnett’s test (B-D) following one-way ANOVA, n.s.p > 0.05, *p < 0.05, **p < 0.01, and ***p < 0.001.
(PDF)

S3 Fig DAF-16 acts independently of CASY-1 and DAF-2c. (A, B) Feeding conditioning with high (A) or low (B) salt, respectively (N = 8). Each red or blue dot represents a chemotaxis index obtained from each trial after conditioning with high or low salt, respectively. Error bars indicate SEM. Tukey’s test following one-way ANOVA, n.s.p > 0.05, **p < 0.01, and ***p < 0.001. (C) Representative images of DAF-2c::Venus expression in the ASER neuron of wild-type (left) and *daf-16* mutant (right) animals. Scale bar indicates 20 μm.
(PDF)

S4 Fig Subcellular localization of DAF-16 and genetic interaction between *daf-2* and *daf-18*. (A) Representative images of DAF-16::GFP in the ASER neuron taken before (naïve) or after conditioning on agar plates containing 100 mM of salt for an hour. Two lines of animals, JN2837 and JN2885, were used. Scale bar indicates 5 μm. (B, C) Representative images (B) and quantification (C) of DAF-16::GFP localization in ASER before (naïve) or after starvation conditioning with 25 mM of salt for an hour. The JN2885 strain was used (N > 15 animals). Scale bar indicates 5 μm. Black and blue dots represent nucleus/cytosol fluorescence intensity ratios in individual animals. Mann Whitney test, ***p < 0.001. (D, E) Feeding conditioning with high (D) or low (E) salt, respectively (N = 6). Each red or blue dot represents a chemotaxis index obtained from each trial after conditioning with high or low salt, respectively. Error bars indicate SEM. Tukey’s test following one-way ANOVA, n.s.p > 0.05, **p < 0.01, and ***p < 0.001.
(PDF)

S5 Fig The effect of DAF-16a(AM) on salt chemotaxis is dependent on Goα and Gqα. (A) Schematic diagram of subcellular localization of wild-type and mutant forms of DAF-16 in ASER. (B) Quantification of DAF-16a::GFP and DAF-16a(AM)::GFP localization in JN2885 and JN2838 animals, respectively (N > 14 animals). Black dots represent nucleus/cytosol fluorescence intensity ratios in individual animals. Mann Whitney test, ***p < 0.001. (C) A schematic diagram of diacylglycerol-dependent signaling in salt chemotaxis. (D) The effect of DAF-16a(AM) on salt chemotaxis after high-salt/feeding conditioning in *goa-1* loss-of-function and *egl-30* gain-of-function mutants.
(PDF)

S6 Fig Salt chemotaxis plasticity in neuropeptide processing enzyme mutants. (A, B) Salt chemotaxis of neuropeptide processing enzyme mutants after starvation conditioning with high (A) or low (B) salt (N = 7 or 8). (C-F) Salt chemotaxis after feeding conditioning with high (C, E) or low (D, F) salt (N = 6 or 7) in neuropeptide processing enzyme mutants (C, D) and *egl-3* transgenic animals (E, F). A red or blue dot represents a chemotaxis index obtained from each trial after conditioning with high or low salt, respectively. Error bars indicate SEM. Dunnett’s test (A-D) and Tukey’s test following one-way ANOVA (E, F). n.s.p > 0.05, *p < 0.05, **p < 0.01, and ***p < 0.001.
(PDF)

S7 Fig DAF-16a(AM) expression in ASER biases chemotaxis towards high salt in some mutants and a transgenic animal. The effect of DAF-16a(AM) expression in ASER on salt chemotaxis after feeding conditioning with low salt in neuropeptide processing enzymes mutants (A), *goa-1* and *egl-30* mutants (B) and interneuron-ablated animals (C) (N = 6 or 7). A blue dot represents a chemotaxis index obtained from each trial after conditioning with low salt. Error bars indicate SEM. Tukey’s test following one-way ANOVA, n.s.p > 0.05, *p < 0.05, and ***p < 0.001.
(PDF)

S8 Fig Expression changes caused by DAF-16a(AM) expression in ASER. (A-F) The expression levels of each gene were examined by qRT-PCR. mRNA was extracted from the wild type and two transgenic lines of animals expressing DAF-16a(AM)::GFP in ASER, namely JN2874 (*daf-16a(AM)* #1) and JN3212 (*daf-16a(AM)* #2). Three biological replicates were used for statistical analysis. An average value of three technical replicates was used to obtain a biological replicate. Error bars indicate SEM. Kruskal-Wallis test, followed by Dunn’s multiple comparison test, * p < 0.05. Note that the differences in *unc-13* expression were not statistically significant (p = 0.0552, Kruskal-Wallis test).
(PDF)

S1 Table *C. elegans* strains used in this study. (PDF)

S2 Table Primers used for cloning of *daf-16* cDNAs and promoter regions. (PDF)

S3 Table Primers used for real-time PCR analyses. (PDF)
