# Supplementary material for: DAF-16/FOXO promotes taste avoidance learning independently of axonal insulin-like signaling
Source: PLoS Genet. 2019 Jul 19;15(7):e1008297. doi: 10.1371/journal.pgen.1008297 (PMC6668909; doi:10.1371/journal.pgen.1008297)
Supplement: S1 Fig — (A) The genomic structures of daf-16 isoforms, and the promoter regions used for expression and behavioral analyses are shown. (B-D) Expression patterns of Venus driven by the promoters of daf-16a, daf-16b, and daf-16f. Venus and each DAF-16 isoform are co-expressed from the venus-fused daf-16 isoform pre-mRNA separated by a sequence with an acceptor site of a spliced leader (SL2) trans-splicing, daf-16::sl2::venus. I, intestine; M, muscle; P, pharynx; S, spermatheca. Scale bars indicate 0.2 mm. (E-G) Expression patterns of the a, b, and f isoforms in the head region. N, neurons; P, pharynx; I, intestine; M, body wall muscle. Scale bar indicates 30 μm. (H-J) ASER expressions of DAF-16 isoforms were confirmed by co-expression of tagRFP driven by the ASER-specific gcy-5 promoter. Scale bar indicates 30 μm. (PDF) [file pgen.1008297.s001.pdf]

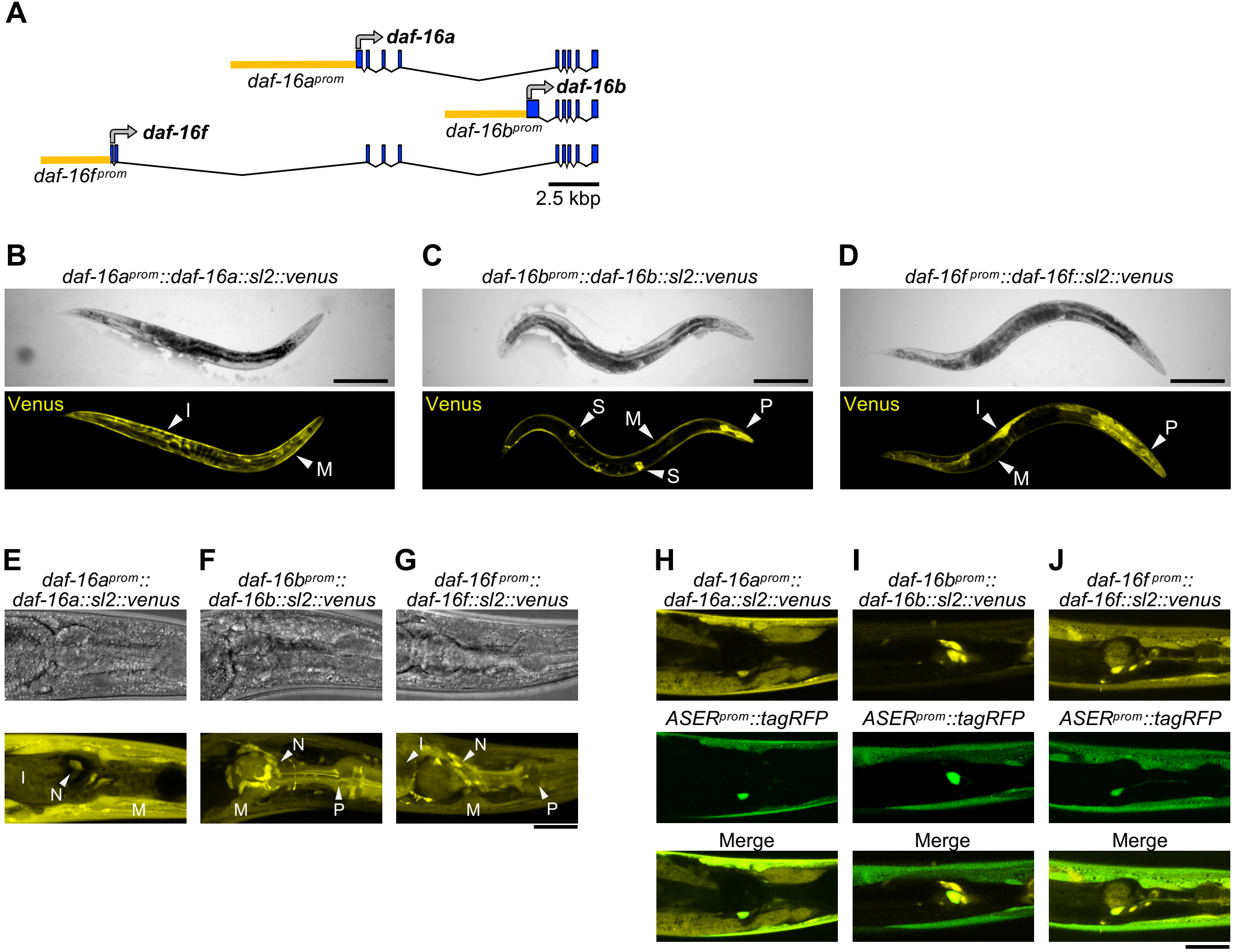

A
daf-16a
daf-16aprom
daf-16b
daf-16bprom
daf-16f
daf-16fprom
2.5 kbp
B
daf-16aprom::daf-16a::sl2::venus
Venus
I
M
C
daf-16bprom::daf-16b::sl2::venus
Venus
S
M
P
S
D
daf-16f prom::daf-16f::sl2::venus
Venus
I
P
M
E
daf-16aprom::
daf-16a::sl2::venus
I
N
M
F
daf-16bprom::
daf-16b::sl2::venus
N
M
P
G
daf-16f prom::
daf-16f::sl2::venus
I
N
M
P
H
daf-16aprom::
daf-16a::sl2::venus
ASERprom::tagRFP
Merge
I
daf-16bprom::
daf-16b::sl2::venus
ASERprom::tagRFP
Merge
J
daf-16f prom::
daf-16f::sl2::venus
ASERprom::tagRFP
Merge
