# Supplementary figures and images for: DAF-16/FOXO promotes taste avoidance learning independently of axonal insulin-like signaling
Source: PLoS Genet. 2019 Jul 19;15(7):e1008297. doi: 10.1371/journal.pgen.1008297 (PMC6668909; doi:10.1371/journal.pgen.1008297)

**A**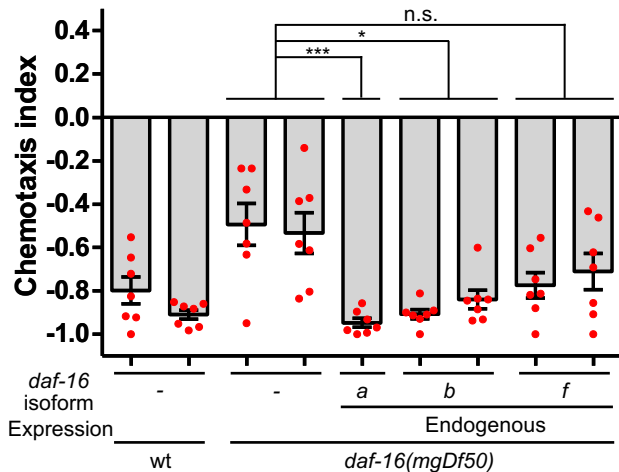**B**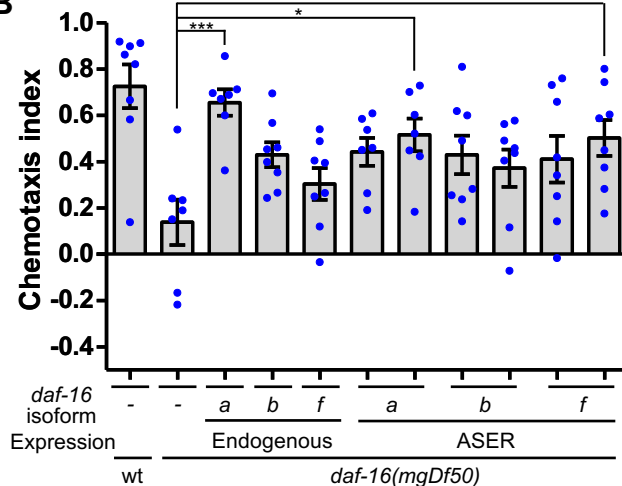**C**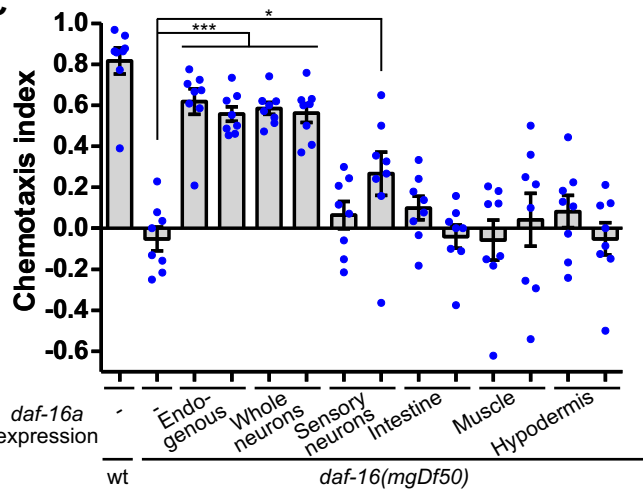**D**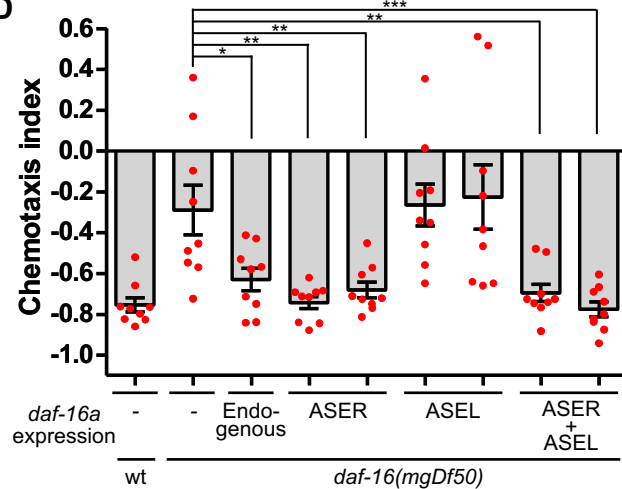

Supplement: S2 Fig — (A-D) Salt chemotaxis after high- (A, D) or low-salt (B, C) conditioning without food in daf-16 transgenic animals. A red or blue dot represents a chemotaxis index obtained from each trial after conditioning with high or low salt, respectively. A repeat of the experiment shown in Fig 2B (A; N = 7). Multiple daf-16 isoform cDNAs were expressed in daf-16(mgDf50) mutant animals by the endogenous promoters or the ASER-specific gcy-5 promoter (B; N = 7 or 8). A DAF-16a isoform was expressed in all or most neurons, amphid and phasmid sensory neurons except ASE and AFD, the intestine, the muscle and the hypodermis of daf-16 mutant animals by the H20, odr-4, ges-1, myo-3, and dpy-7 promoters, respectively (C; N = 8). (C, D) Salt chemotaxis after high-salt conditioning without food in daf-16 transgenic animals. daf-16a::gfp was expressed in daf-16 mutant animals under the endogenous promoter, the ASER-specific gcy-5 promoter or the ASEL-specific gcy-7 promoter (D; N = 9). Error bars indicate SEM. Tukey’s test (A) or Dunnett’s test (B-D) following one-way ANOVA, n.s.p > 0.05, *p < 0.05, **p < 0.01, and ***p < 0.001. (PDF) [file pgen.1008297.s002.pdf]

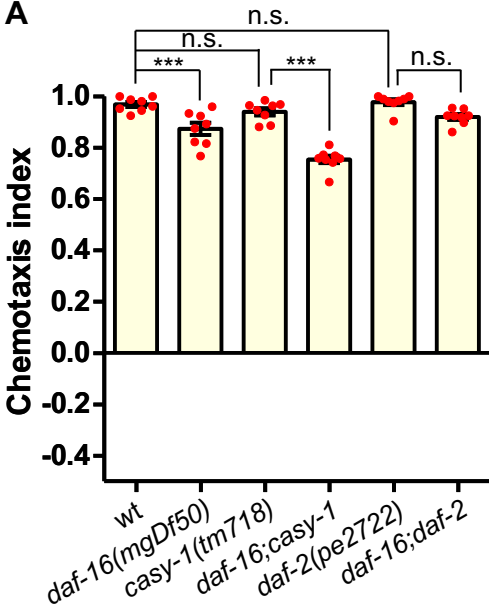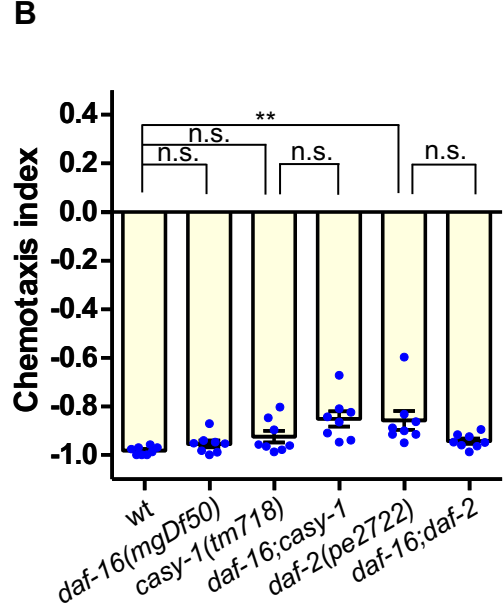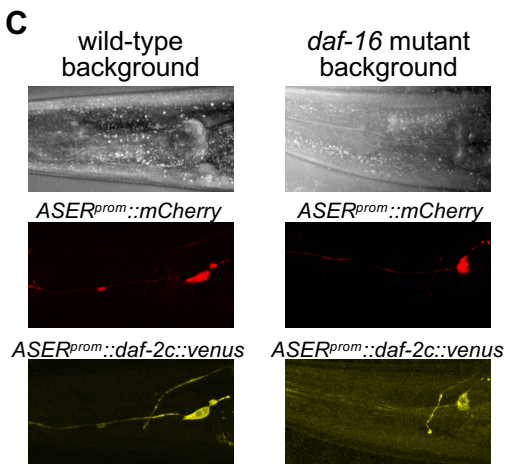

Supplement: S3 Fig — (A, B) Feeding conditioning with high (A) or low (B) salt, respectively (N = 8). Each red or blue dot represents a chemotaxis index obtained from each trial after conditioning with high or low salt, respectively. Error bars indicate SEM. Tukey’s test following one-way ANOVA, n.s.p > 0.05, **p < 0.01, and ***p < 0.001. (C) Representative images of DAF-2c::Venus expression in the ASER neuron of wild-type (left) and daf-16 mutant (right) animals. Scale bar indicates 20 μm. (PDF) [file pgen.1008297.s003.pdf]

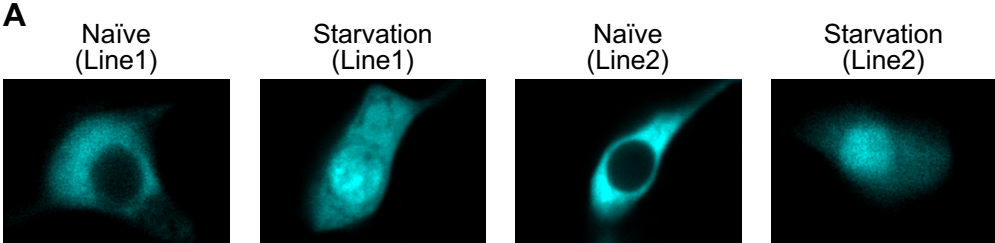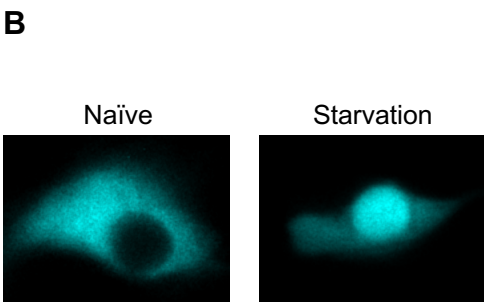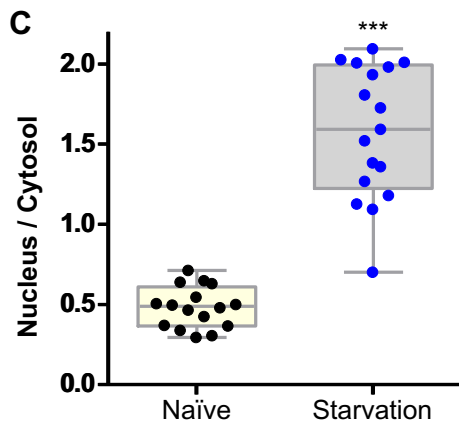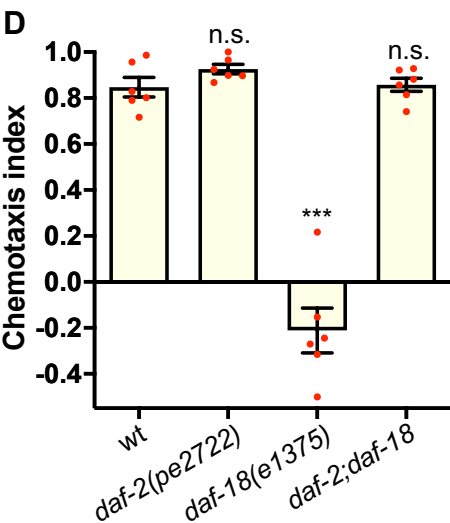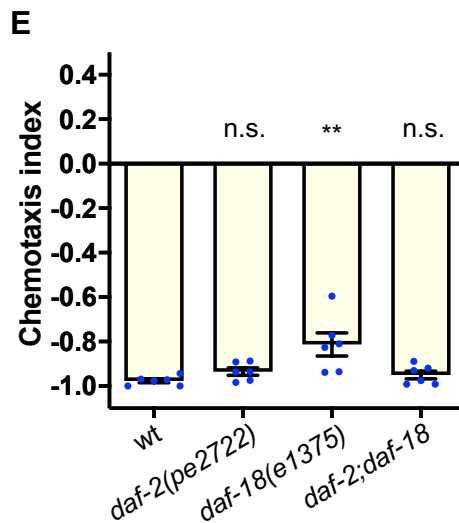

Supplement: S4 Fig — (A) Representative images of DAF-16::GFP in the ASER neuron taken before (naïve) or after conditioning on agar plates containing 100 mM of salt for an hour. Two lines of animals, JN2837 and JN2885, were used. Scale bar indicates 5 μm. (B, C) Representative images (B) and quantification (C) of DAF-16::GFP localization in ASER before (naïve) or after starvation conditioning with 25 mM of salt for an hour. The JN2885 strain was used (N > 15 animals). Scale bar indicates 5 μm. Black and blue dots represent nucleus/cytosol fluorescence intensity ratios in individual animals. Mann Whitney test, ***p < 0.001. (D, E) Feeding conditioning with high (D) or low (E) salt, respectively (N = 6). Each red or blue dot represents a chemotaxis index obtained from each trial after conditioning with high or low salt, respectively. Error bars indicate SEM. Tukey’s test following one-way ANOVA, n.s.p > 0.05, **p < 0.01, and ***p < 0.001. (PDF) [file pgen.1008297.s004.pdf]

**A**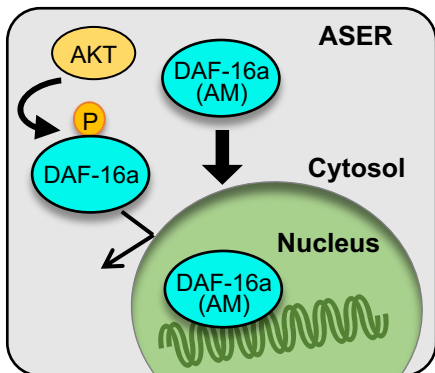**B**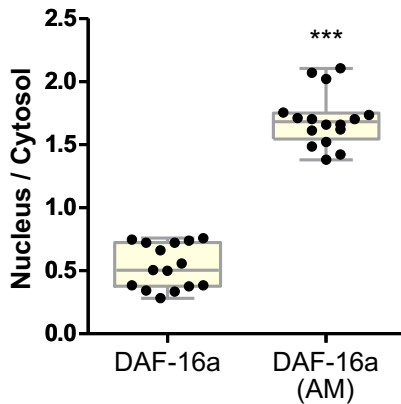**C**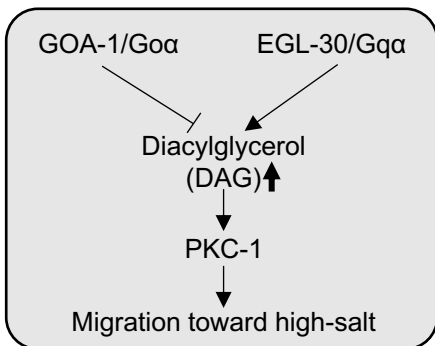**D**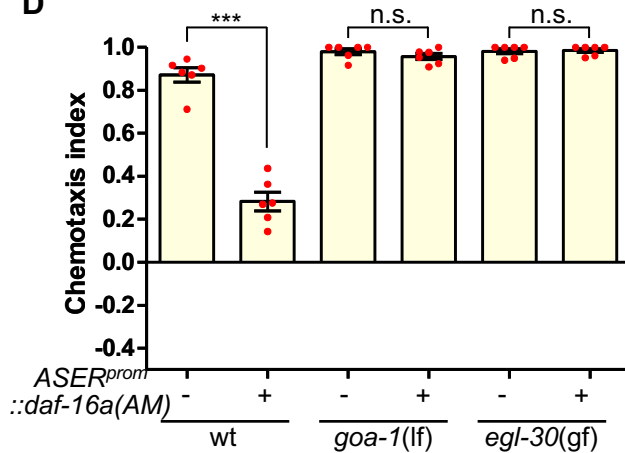

Supplement: S5 Fig — (A) Schematic diagram of subcellular localization of wild-type and mutant forms of DAF-16 in ASER. (B) Quantification of DAF-16a::GFP and DAF-16a(AM)::GFP localization in JN2885 and JN2838 animals, respectively (N > 14 animals). Black dots represent nucleus/cytosol fluorescence intensity ratios in individual animals. Mann Whitney test, ***p < 0.001. (C) A schematic diagram of diacylglycerol-dependent signaling in salt chemotaxis. (D) The effect of DAF-16a(AM) on salt chemotaxis after high-salt/feeding conditioning in goa-1 loss-of-function and egl-30 gain-of-function mutants. (PDF) [file pgen.1008297.s005.pdf]

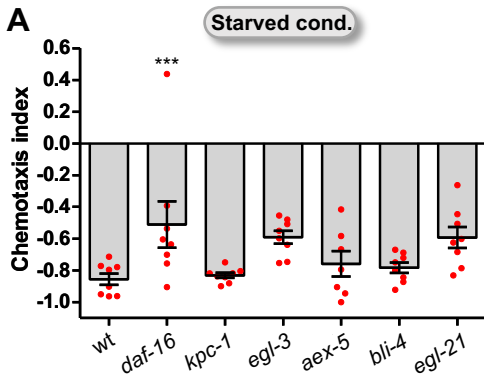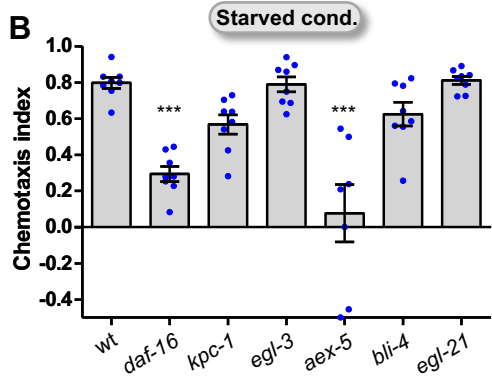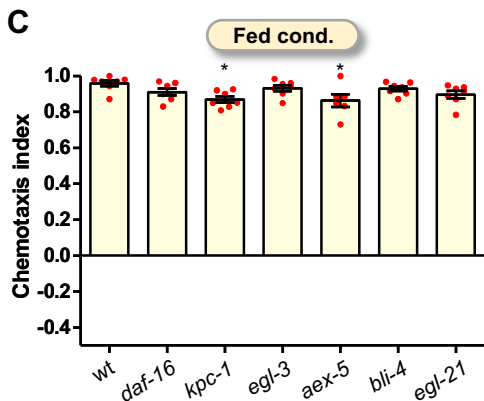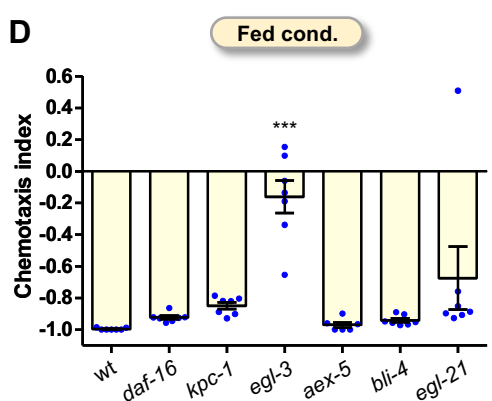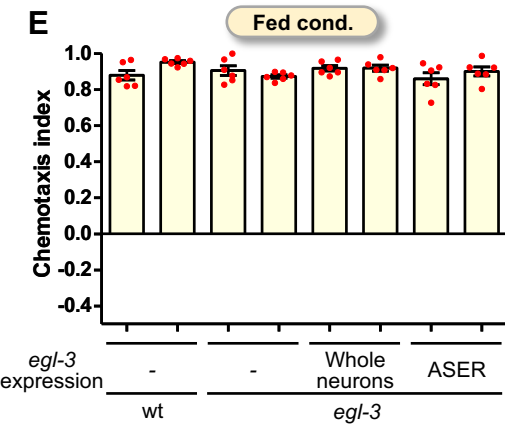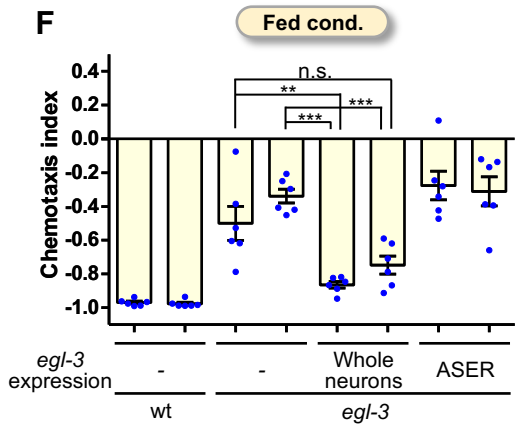

Supplement: S6 Fig — (A, B) Salt chemotaxis of neuropeptide processing enzyme mutants after starvation conditioning with high (A) or low (B) salt (N = 7 or 8). (C-F) Salt chemotaxis after feeding conditioning with high (C, E) or low (D, F) salt (N = 6 or 7) in neuropeptide processing enzyme mutants (C, D) and egl-3 transgenic animals (E, F). A red or blue dot represents a chemotaxis index obtained from each trial after conditioning with high or low salt, respectively. Error bars indicate SEM. Dunnett’s test (A-D) and Tukey’s test following one-way ANOVA (E, F). n.s.p > 0.05, *p < 0.05, **p < 0.01, and ***p < 0.001. (PDF) [file pgen.1008297.s006.pdf]

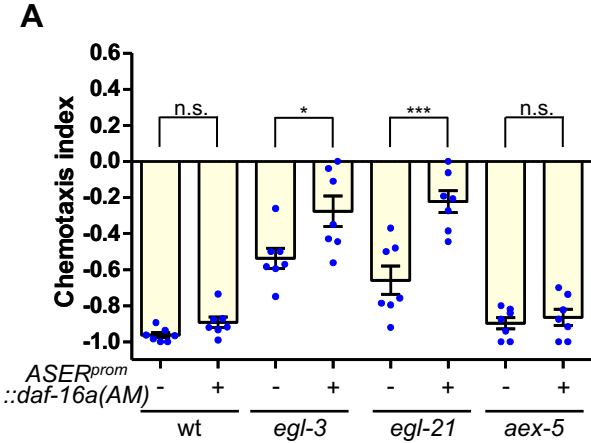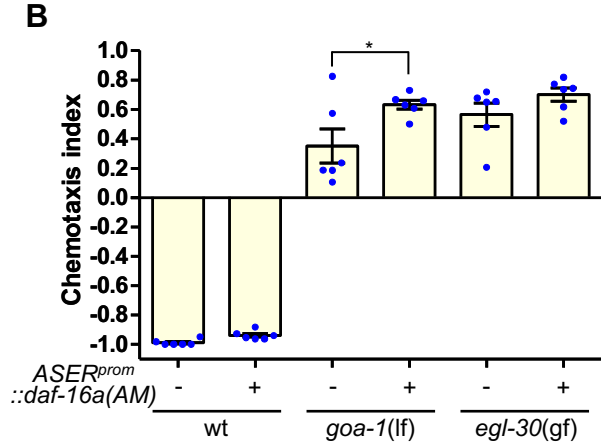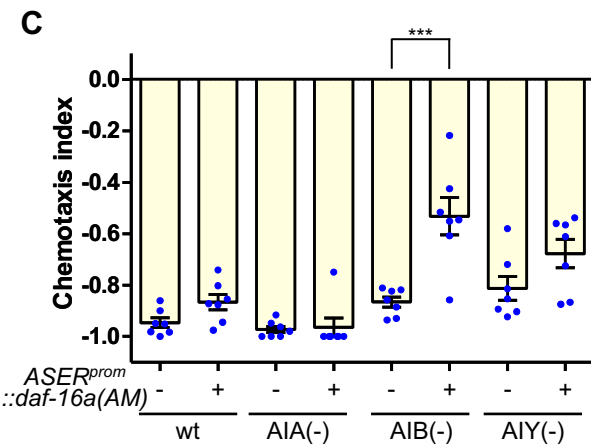

Supplement: S7 Fig — The effect of DAF-16a(AM) expression in ASER on salt chemotaxis after feeding conditioning with low salt in neuropeptide processing enzymes mutants (A), goa-1 and egl-30 mutants (B) and interneuron-ablated animals (C) (N = 6 or 7). A blue dot represents a chemotaxis index obtained from each trial after conditioning with low salt. Error bars indicate SEM. Tukey’s test following one-way ANOVA, n.s.p > 0.05, *p < 0.05, and ***p < 0.001. (PDF) [file pgen.1008297.s007.pdf]

**A*****egl-3***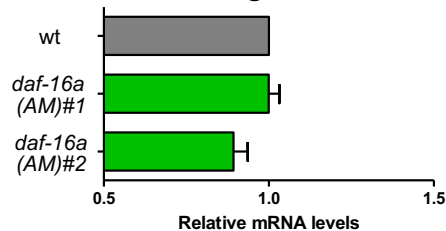**B*****egl-21***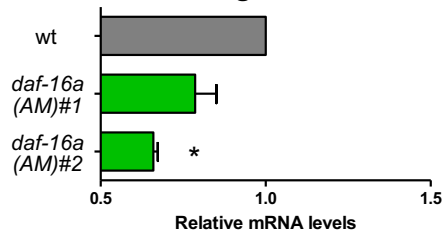**C*****pkc-1***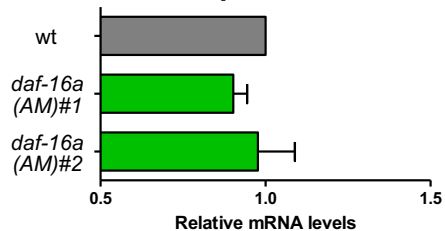**D*****unc-13***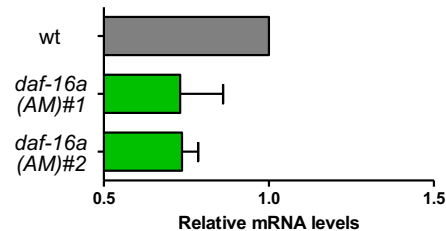**E*****eat-4***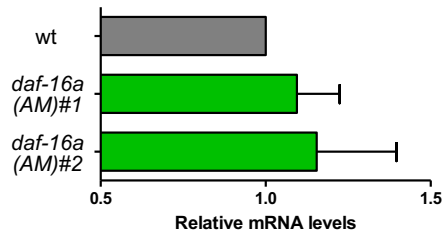**F*****dgk-1***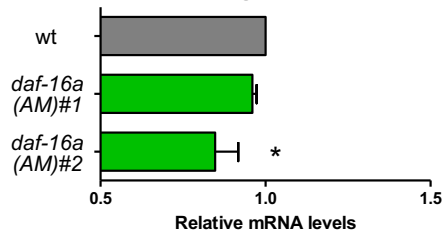

Supplement: S8 Fig — (A-F) The expression levels of each gene were examined by qRT-PCR. mRNA was extracted from the wild type and two transgenic lines of animals expressing DAF-16a(AM)::GFP in ASER, namely JN2874 (daf-16a(AM) #1) and JN3212 (daf-16a(AM) #2). Three biological replicates were used for statistical analysis. An average value of three technical replicates was used to obtain a biological replicate. Error bars indicate SEM. Kruskal-Wallis test, followed by Dunn’s multiple comparison test, * p < 0.05. Note that the differences in unc-13 expression were not statistically significant (p = 0.0552, Kruskal-Wallis test). (PDF) [file pgen.1008297.s008.pdf]
