# Supplementary material for: DAF-16/FOXO promotes taste avoidance learning independently of axonal insulin-like signaling
Source: PLoS Genet. 2019 Jul 19;15(7):e1008297. doi: 10.1371/journal.pgen.1008297 (PMC6668909; doi:10.1371/journal.pgen.1008297)
Supplement: S1 Table — (PDF) [file pgen.1008297.s009.pdf]

Strain name	Genotype	Source	Used for
N2	wild-type	CGC	Learning test
GR1307	daf-16(mgDf50) I.	CGC	
JN714	daf-16(mgDf47) I.	Tomioka *et al.*, 2006	
DR26	daf-16(m26) I.	CGC	
FX05030	daf-16(tm5030) I.	NBRP	
FX05031	daf-16(tm5031) I.	NBRP	
FX06659	daf-16(tm6659) I.	NBRP	
CB937	bli-4(e937) I.	CGC	
VC48	kpc-1(gk8) I.	CGC	
JT23	aex-5(sa23) I.	CGC	
JN433	casy-1(tm718) II.	Ikeda *et al.*, 2008	
JN3213	daf-16(mgDf50) I; casy-1(tm718) II.	This study	
JN2722	daf-2(pe2722) III.	This study	
JN3214	daf-16(mgDf50) I; daf-2(pe2722) III.	This study	
JN146	daf-18(ok480) IV.	This study	
JN2843	daf-16(mgDf50) I; daf-18(ok480) IV.	This study	
JN709	daf-18(mg198) IV.	Tomioka *et al.*, 2006	
JN2844	daf-16(mgDf50) I; daf-18(mg198) IV.	This study	
CB1375	daf-18(e1375)	CGC	
JN2760	daf-2(pe2722); daf-18(e1375)	This study	
KP2018	egl-21(n476) IV.	CGC	
VC461	egl-3(gk238) V.	CGC	
IK130	pkc-1(nj3) V.	From I. Mori	
JN2824	Ex[myo-3^{prom}::mCherry].	This study	Learning test (daf-16 rescue)
JN2825	daf-16(mgDf50) I; Ex[myo-3^{prom}::mCherry].	This study	
JN2827	daf-16(mgDf50) I; Ex[daf-16a^{prom}::daf-16a::sl2::venus, Pmyo-3^{prom}::mCherry]. line1	This study	
-	daf-16(mgDf50) I; Ex[daf-16a^{prom}::daf-16a::sl2::venus, Pmyo-3^{prom}::mCherry]. line2	This study	
JN2828	daf-16(mgDf50) I; Ex[daf-16b^{prom}::daf-16b::sl2::venus, Pmyo-3^{prom}::mCherry].	This study	
JN2829	daf-16(mgDf50) I; Ex[daf-16f^{prom}::daf-16f::sl2::venus, Pmyo-3^{prom}::mCherry].	This study	
JN2830	daf-16(mgDf50) I; Ex[gcy-5^{prom}::daf-16a::sl2::venus, myo-3^{prom}::mCherry].	This study	
JN2831	daf-16(mgDf50) I; Ex[gcy-5^{prom}::daf-16b::sl2::venus, myo-3^{prom}::mCherry].	This study	
JN2832	daf-16(mgDf50) I; Ex[gcy-5^{prom}::daf-16f::sl2::venus, myo-3^{prom}::mCherry].	This study	
JN3217	daf-16(mgDf50) I; Ex[H20^{prom}::daf-16a::sl2::venus, myo-3^{prom}::mCherry]. line1	This study	
-	daf-16(mgDf50) I; Ex[H20^{prom}::daf-16a::sl2::venus, myo-3^{prom}::mCherry]. line2	This study	
JN3218	daf-16(mgDf50) I; Ex[odr-4^{prom}::daf-16a::sl2::venus, myo-3^{prom}::mCherry]. line1	This study	
-	daf-16(mgDf50) I; Ex[odr-4^{prom}::daf-16a::sl2::venus, myo-3^{prom}::mCherry]. line2	This study	
JN3219	daf-16(mgDf50) I; Ex[ges-1^{prom}::daf-16a::sl2::venus, myo-3^{prom}::mCherry]. line1	This study	
-	daf-16(mgDf50) I; Ex[ges-1^{prom}::daf-16a::sl2::venus, myo-3^{prom}::mCherry]. line2	This study	
JN3220	daf-16(mgDf50) I; Ex[myo-3^{prom}::daf-16a::sl2::venus, myo-3^{prom}::mCherry]. line1	This study	
-	daf-16(mgDf50) I; Ex[myo-3^{prom}::daf-16a::sl2::venus, myo-3^{prom}::mCherry]. line2	This study	
JN3221	daf-16(mgDf50) I; Ex[dpy-7^{prom}::daf-16a::sl2::venus, myo-3^{prom}::mCherry]. line1	This study	
-	daf-16(mgDf50) I; Ex[dpy-7^{prom}::daf-16a::sl2::venus, myo-3^{prom}::mCherry]. line2	This study	
JN798	Ex[myo-3^{prom}::gfp].	This study	Learning test (daf-16a::gfp rescue)
JN3231	daf-16(mgDf50) I; Ex[myo-3^{prom}::gfp].	This study	
JN3232	daf-16(mgDf50) I; Ex[daf-16a^{prom}::daf-16a::gfp, myo-3^{prom}::gfp].	This study	
JN3233	daf-16(mgDf50) I; Ex[gcy-5^{prom}::daf-16a::gfp, myo-3^{prom}::gfp]. line1	This study	
-	daf-16(mgDf50) I; Ex[gcy-5^{prom}::daf-16a::gfp, myo-3^{prom}::gfp]. line2	This study	
JN3234	daf-16(mgDf50) I; Ex[gcy-7^{prom}::daf-16a::gfp, myo-3^{prom}::gfp]. line1	This study	
-	daf-16(mgDf50) I; Ex[gcy-7^{prom}::daf-16a::gfp, myo-3^{prom}::gfp]. line2	This study	
JN3235	daf-16(mgDf50) I; Ex[gcy-5^{prom}::daf-16a::gfp, gcy-7^{prom}::daf-16a::gfp, myo-3^{prom}::gfp]. line1	This study	
-	daf-16(mgDf50) I; Ex[gcy-5^{prom}::daf-16a::gfp, gcy-7^{prom}::daf-16a::gfp, myo-3^{prom}::gfp]. line2	This study	
JN3203	Ex[gcy-5^{prom}::TIR1::sl2::cfp, myo-3^{prom}::mCherry]. line1	This study	Learning test (auxin-degron)
JN3204	Ex[gcy-5^{prom}::TIR1::sl2::cfp, myo-3^{prom}::mCherry]. line2	This study	
JN3205	daf-16(mgDf50) I; Ex[gcy-5^{prom}::TIR1::sl2::cfp, myo-3^{prom}::mCherry]. line1	This study	
JN3206	daf-16(mgDf50) I; Ex[gcy-5^{prom}::TIR1::sl2::cfp, myo-3^{prom}::mCherry]. line2	This study	
JN3207	daf-16(mgDf50) I; Ex[gcy-5^{prom}::TIR1::sl2::cfp, gcy-5^{prom}::degron::daf-16a::gfp, myo-3^{prom}::mCherry]. line1	This study	
JN3208	daf-16(mgDf50) I; Ex[gcy-5^{prom}::TIR1::sl2::cfp, gcy-5^{prom}::degron::daf-16a::gfp, myo-3^{prom}::mCherry]. line2	This study	
JN2817	Ex[myo-3^{prom}::venus]. line1	This study	Learning test (daf-16(AM))
-	Ex[myo-3^{prom}::venus]. line2	This study	
JN2816	Ex[gcy-5^{prom}::daf-16a(AM)::gfp, myo-3^{prom}::venus]. line1	This study	
-	Ex[gcy-5^{prom}::daf-16a(AM)::gfp, myo-3^{prom}::venus]. line2	This study	
JN2835	daf-16(mgDf50) I; Ex[myo-3^{prom}::venus]. line1	This study	
-	daf-16(mgDf50) I; Ex[myo-3^{prom}::venus]. line2	This study	
JN2836	daf-16(mgDf50) I; Ex[gcy-5^{prom}::daf-16a(AM)::gfp, myo-3^{prom}::venus]. line1	This study	
-	daf-16(mgDf50) I; Ex[gcy-5^{prom}::daf-16a(AM)::gfp, myo-3^{prom}::venus]. line2	This study	
JN3216	daf-16(mgDf50) I; Ex[gcy-7^{prom}::daf-16a(AM)::gfp, myo-3^{prom}::venus]. line1	This study	
-	daf-16(mgDf50) I; Ex[gcy-7^{prom}::daf-16a(AM)::gfp, myo-3^{prom}::venus]. line2	This study	
JN2880	egl-30(pe914) I; Ex[myo-3^{prom}::venus].	This study	
JN2881	egl-30(pe914) I; Ex[gcy-5^{prom}::daf-16a(AM)::gfp, myo-3^{prom}::venus].	This study	
JN2878	goa-1(n1134) I; Ex[myo-3^{prom}::venus].	This study	
JN2879	goa-1(n1134) I; Ex[gcy-5^{prom}::daf-16a(AM)::gfp, myo-3^{prom}::venus].	This study	
JN2854	aex-5(sa23) I; Ex[myo-3^{prom}::venus].	This study	
JN2855	aex-5(sa23) I; Ex[gcy-5^{prom}::daf-16a(AM)::gfp, myo-3^{prom}::venus].	This study	
JN3223	casy-1(tm718) II; Ex[myo-3^{prom}::venus].	This study	
JN3224	casy-1(tm718) II; Ex[gcy-5^{prom}::daf-16a(AM)::gfp, myo-3^{prom}::venus].	This study	
JN3225	daf-2(pe2722) III; Ex[myo-3^{prom}::venus].	This study	
JN3226	daf-2(pe2722) III; Ex[gcy-5^{prom}::daf-16a(AM)::gfp, myo-3^{prom}::venus].	This study	
JN2856	egl-21(n476) IV; Ex[myo-3^{prom}::venus].	This study	
JN2857	egl-21(n476) IV; Ex[gcy-5^{prom}::daf-16a(AM)::gfp, myo-3^{prom}::venus].	This study	
JN2834	egl-3(gk238) V; Ex[myo-3^{prom}::venus].	This study	
JN2833	egl-3(gk238) V; Ex[gcy-5^{prom}::daf-16a(AM)::gfp, myo-3^{prom}::venus].	This study	
JN2821	pkc-1(nj3) V; Ex[myo-3^{prom}::venus].	This study	
JN2820	pkc-1(nj3) V; Ex[gcy-5^{prom}::daf-16a(AM)::gfp, myo-3^{prom}::venus].	This study	
JN3227	peIs580[ins-1(short)^{prom}::casp1, ins-1(short)^{prom}::venus, unc-122^{prom}::gfp]; Ex[gcy-5^{prom}::daf-16(AM)::gfp, myo-3^{prom}::venus].	This study	
JN3228	peIs578[npr-9^{prom}::casp1, npr-9^{prom}::venus, unc-122^{prom}::mCherry]; Ex[gcy-5^{prom}::daf-16(AM)::gfp, myo-3^{prom}::venus].	This study	
JN3229	peIs579[ttx-3^{prom}::casp1, ttx-3^{prom}::venus, lin-44^{prom}::gfp]; Ex[gcy-5^{prom}::daf-16(AM)::gfp, myo-3^{prom}::venus].	This study	
JN2834	egl-3(gk238) V; Ex[myo-3^{prom}::venus].	This study	Learning test (egl-3 rescue)
JN2833	egl-3(gk238) V; Ex[gcy-5^{prom}::daf-16a(AM)::gfp, myo-3^{prom}::venus].	This study	
JN2883	egl-3(gk238) V; Ex[H20^{prom}::egl-3, myo-3^{prom}::venus]. line1	This study	
-	egl-3(gk238) V; Ex[H20^{prom}::egl-3, myo-3^{prom}::venus]. line2	This study	
JN2884	egl-3(gk238) V; Ex[gcy-5^{prom}::egl-3, myo-3^{prom}::venus]. line1	This study	
-	egl-3(gk238) V; Ex[gcy-5^{prom}::egl-3, myo-3^{prom}::venus]. line2	This study	
JN2896	Ex[unc-122^{prom}::mCherry].	This study	
JN2898	egl-3(gk238) V; Is[gcy-5^{prom}::daf-16a(AM)::gfp, myo-3^{prom}::venus]; Ex[unc-122^{prom}::mCherry]. line1	This study	
-	egl-3(gk238) V; Is[gcy-5^{prom}::daf-16a(AM)::gfp, myo-3^{prom}::venus]; Ex[unc-122^{prom}::mCherry]. line2	This study	
JN2899	egl-3(gk238) V; Is[gcy-5^{prom}::daf-16a(AM)::gfp, myo-3^{prom}::venus]; Ex[H20^{prom}::egl-3, unc-122^{prom}::mCherry]. line1	This study	
-	egl-3(gk238) V; Is[gcy-5^{prom}::daf-16a(AM)::gfp, myo-3^{prom}::venus]; Ex[H20^{prom}::egl-3, unc-122^{prom}::mCherry]. line2	This study	
JN3268	Ex[gcy-5^{prom}::egl-21::sl2::cfp, myo-3^{prom}::mCherry]. line1	This study	Learning test (egl-21)
JN3269	Ex[gcy-5^{prom}::egl-21::sl2::cfp, myo-3^{prom}::mCherry]. line2	This study	
JN2874	Is[gcy-5^{prom}::daf-16a(AM)::gfp, myo-3^{prom}::venus]. line1	This study	Learning test and qPCR
JN3212	Is[gcy-5^{prom}::daf-16a(AM)::gfp, myo-3^{prom}::venus]. line2	This study	
JN2810	daf-16(mgDf50) I; Ex[daf-16a^{prom}::daf-16a::sl2::venus, gcy-5^{prom}::tagRFP, myo-3^{prom}::mCherry].	This study	Isoform expression
JN2812	daf-16(mgDf50) I; Ex[daf-16b^{prom}::daf-16b::sl2::venus, gcy-5^{prom}::tagRFP, myo-3^{prom}::mCherry].	This study	
JN2814	daf-16(mgDf50) I; Ex[daf-16f^{prom}::daf-16f::sl2::venus, gcy-5^{prom}::tagRFP, myo-3^{prom}::mCherry].	This study	
JN2837	Is[H20^{prom}::nls4::mCherry]; Ex[gcy-5^{prom}::daf-16a::gfp, gcy-5^{prom}::tagRFP, lin-44^{prom}::venus]. line1	This study	DAF-16 localization
JN2885	Is[H20^{prom}::nls4::mCherry]; Ex[gcy-5^{prom}::daf-16a::gfp, gcy-5^{prom}::tagRFP, lin-44^{prom}::venus]. line2	This study	
JN2838	Is[H20^{prom}::nls4::mCherry]; Ex[gcy-5^{prom}::daf-16a(AM)::gfp, gcy-5^{prom}::tagRFP, lin-44^{prom}::venus].	This study	
JN3210	daf-2(e1370) III; Is[H20^{prom}::nls4::mCherry]; Ex[gcy-5^{prom}::daf-16a::gfp, gcy-5^{prom}::tagRFP, lin-44^{prom}::venus].	This study	
JN3209	daf-2(pe2722) III; Is[H20^{prom}::nls4::mCherry]; Ex[gcy-5^{prom}::daf-16a::gfp, gcy-5^{prom}::tagRFP, lin-44^{prom}::venus].	This study	
JN3271	daf-2(e1370) III; Is[H20^{prom}::nls4::mCherry]; Ex[gcy-5^{prom}::daf-16a::gfp, gcy-5^{prom}::tagRFP, lin-44^{prom}::venus, gcy-5^{prom}::daf-2a, unc-122^{prom}::mCherry].	This study	
JN1524	Is[gcy-5^{prom}::daf-2c::venus, unc122^{prom}::mCherry]; Is[gcy-5^{prom}::mCherry, lin-44^{prom}::GFP].	Ohno *et al.*, 2014	DAF-2c localization
JN3236	daf-16(mgDf50) I; Is[gcy-5^{prom}::daf-2c::venus, unc122^{prom}::mCherry]; Is[gcy-5^{prom}::mCherry, lin-44^{prom}::GFP].	This study	
