# Supplementary material for: DAF-16/FOXO promotes taste avoidance learning independently of axonal insulin-like signaling
Source: PLoS Genet. 2019 Jul 19;15(7):e1008297. doi: 10.1371/journal.pgen.1008297 (PMC6668909; doi:10.1371/journal.pgen.1008297)
Supplement: S2 Table — (PDF) [file pgen.1008297.s010.pdf]

Primer name	Sequence (5' > 3')
daf-16a_cDNA_Fw	cgaaGCTAGCatgatggagatgctggtag
daf-16a_cDNA_Rv	gaaGGTACCttacaaatcaaaatgaatatg
daf-16b_cDNA_Fw	agctGCTAGCatgaacgactcaatagac
daf-16b_cDNA_Rv	tgaaGGTACCttacaaatcaaaatg
daf-16f_cDNA_Fw	ctcaGCTAGCatgcaagcgtggaactg
daf-16f_cDNA_Rv	gaaGGTACCttacaaatcaaaatgaatatgc
daf-16a_promoter_Fw	gatcGGATCCgaattcaacttgagc
daf-16a_promoter_Rv	ggggGCGGCCGCagtgagttctctgga
daf-16b_promoter_Fw	ctagGGATCCtactggaagccaacgg
daf-16b_promoter_Rv	tgagGCGGCCGCtctggaagctgtgctc
daf-16f_promoter_Fw	aattGGATCCgagagacggctcgaaaag
daf-16f_promoter_Rv	ccacGCGGCCGCgtcctgtgagaattttg
