# Supplementary material for: DAF-16/FOXO promotes taste avoidance learning independently of axonal insulin-like signaling
Source: PLoS Genet. 2019 Jul 19;15(7):e1008297. doi: 10.1371/journal.pgen.1008297 (PMC6668909; doi:10.1371/journal.pgen.1008297)
Supplement: S3 Table — (PDF) [file pgen.1008297.s011.pdf]

Target gene	Sequence (5' > 3')
eft-3	ATTGCCACACCGCTCACA<br>GGGAAGTCCTCAACCTTCTTAC
egl-3	TGACGGAGTGGATTACATGC<br>TCCGTGGGAATTGAACCAGTC
egl-21	ACTTGAAGCAAAGTTGGGAG<br>ATGCTCTCCAGGAGTTGTCG
pkc-1	TATTCTTGCGTCAGCCAACG<br>GGCAACGTTTATGAACTACGAC
unc-13	GCAGTCTCTGCTCCTGATAG<br>TTGAAGTTTGGCAGGCGACG
eat-4	TCATACGTCTCATGGGCTGC<br>TTGCGATATGGTCGGATGAAAC
dgk-1	GGATGGGTACTGCAATGTTTAG<br>CGGAGAACTCTTGCCAAATCG
